# A modular deep learning architecture for interpretable disease prediction across tabular clinical and biometric datasets

**DOI:** 10.1371/journal.pone.0348670

**Published:** 2026-05-08

**Authors:** Vijay U. Rathod, Siddhesh Sanjay Amrutkar, Kirti A. Patil, Aoudumber D. Londhe, Sandip Y. Bobade, Virendrakumar A. Dhotre, Melkamu Workie Kebede

**Affiliations:** 1 Department of CSE (Artificial Intelligence and Machine Learning), Vishwakarma Institute of Technology (Affiliated to Savitribai Phule Pune University, Pune), Pune, Maharashtra, India; 2 Department of Information Technology, MET’s Institute of Engineering (Affiliated to Savitribai Phule Pune University, Pune), Nashik, Maharashtra, India; 3 Department of Artificial Intelligence and Data Science, Vishwakarma Institute of Technology (Affiliated to Savitribai Phule Pune University Pune), Pune, Maharashtra, India; 4 Department of Computer Science and Engineering (Artificial Intelligence), Vishwakarma Institute of Technology (Affiliated to Savitribai Phule Pune University Pune), Pune, Maharashtra, India; 5 Department of Computer Science and Engineering (Artificial Intelligence and Machine Learning), Vishwakarma Institute of Technology (Affiliated to Savitribai Phule Pune University, Pune), Pune, Maharashtra, India; 6 Department of Information Technology, Institute of Technology, Debre Markos University, Debre Markos, Ethiopia; Incheon National University, KOREA, REPUBLIC OF

## Abstract

Accurate disease prediction using clinical datasets is essential for improving early diagnosis and clinical decision-support systems; however, many existing deep learning approaches are disease-specific, computationally intensive, and difficult to generalize across heterogeneous biomedical datasets. This study addresses this challenge by proposing a unified and dataset-aware deep learning framework that enables accurate and interpretable disease prediction across diverse clinical datasets. The framework adopts a modular architecture that selects appropriate models based on dataset characteristics such as feature dimensionality, sample size, and class imbalance. It integrates multiple deep learning architectures, including MLP, one-dimensional CNN, FT-Transformer, autoencoder-based classifiers, and ensemble strategies. Robust preprocessing, fold-safe feature selection, and nested cross-validation are incorporated to ensure reliable performance evaluation. The framework is evaluated on three heterogeneous benchmark datasets: the UCI Heart Disease dataset (303 samples, 13 clinical features), the PIMA Indians Diabetes dataset (768 samples, 8 metabolic features), and the Parkinson’s disease voice dataset (195 recordings, 22 acoustic features). Experimental results demonstrate competitive predictive performance relative to classical baselines across the diverse tasks. The FT-Transformer + autoencoder ensemble achieved an AUC of 0.8980 (±0.0483) for heart disease prediction, while the CNN + Autoencoder ensemble obtained an AUC of 0.8451 (±0.0270) for diabetes classification. For Parkinson’s disease detection, the MLP achieved an AUC of 0.7538 with perfect specificity. Overall, all models achieved AUC values comparable to ML baselines. The study contributes a scalable and interpretable deep learning framework that improves reliability, generalization, and practical applicability for multi-disease prediction in real-world healthcare environments.

## Introduction

In the past decade, substantial research efforts have been devoted to developing deep learning–based models for disease prediction using clinical data, driven by the growing availability of electronic health records and advances in computational intelligence. Existing studies have demonstrated that deep neural networks, including convolutional, recurrent, and attention-based architectures, can achieve high predictive accuracy across a range of medical conditions [1]. However, much of this research has focused on disease-specific or dataset-specific models, often resulting in architectures that are computationally intensive, difficult to interpret, and tightly coupled to curated datasets. Such limitations hinder generalizability across heterogeneous clinical environments and restrict practical deployment in real-world healthcare settings, particularly where computational resources and model transparency are critical [2].

More recently, research trends have begun to emphasize the need for interpretable and lightweight unified deep learning frameworks capable of maintaining high predictive performance while addressing heterogeneity in clinical datasets. These approaches aim to balance model complexity, interpretability, and robustness by incorporating modular architectures, attention mechanisms, and efficient representation learning strategies. Despite promising progress, existing frameworks frequently lack systematic dataset-aware adaptation and consistent evaluation protocols across multiple disease domains [3]. This gap underscores the need for an integrated, scalable, and interpretable unified framework that can accurately predict diverse diseases from heterogeneous clinical data while remaining suitable for real-time clinical decision support and deployment in resource-constrained healthcare systems.

Despite extensive progress in medical deep learning, the problem of designing an interpretable and lightweight unified deep learning framework for accurate disease prediction across heterogeneous clinical datasets remains insufficiently resolved due to several fundamental challenges. Clinical datasets differ significantly in terms of feature dimensionality, data distributions, modality types, class imbalance, and sample sizes, making it difficult to develop a single framework that generalizes effectively without extensive dataset-specific tuning. Many existing models achieve high accuracy by increasing architectural complexity, which leads to large parameter counts, high computational cost, and reduced transparency. As a result, these models often function as black boxes, limiting clinical trust and interpretability, and are impractical for deployment in real-world healthcare environments, particularly in resource-constrained settings [4].

Despite significant advancements in medical deep learning, most existing studies focus on disease-specific models designed for single datasets or narrowly defined prediction tasks. These approaches often lack generalizability across heterogeneous clinical datasets with varying feature structures, sample sizes, and data distributions. Furthermore, many high-performing deep learning models emphasize accuracy while neglecting interpretability, computational efficiency, and methodological consistency. The absence of a unified, dataset-aware deep learning framework that can systematically adapt to different clinical datasets represents a major gap in the current literature [5].

This study primarily focuses on designing a modular and dataset-aware deep learning framework capable of adapting its architecture based on dataset characteristics. The proposed approach incorporates consistent preprocessing, feature standardization, attention mechanisms, and ensemble learning strategies to improve predictive reliability. The framework is evaluated on three heterogeneous biomedical datasets, including the UCI Heart Disease dataset (303 samples, 13 features), the PIMA Indians Diabetes dataset (768 samples, 8 features), and a Parkinson’s disease voice dataset (195 samples). Experimental results demonstrate competitive predictive performance, achieving AUC values comparable to established classical machine learning baselines across the datasets.

The primary aim of this study is to develop a dataset-aware framework for accurate disease prediction across diverse clinical datasets. Rather than proposing novel individual architectures, this study systematically integrates them and contributes: 1. A dataset-aware benchmarking framework for deep learning on heterogeneous clinical tabular datasets. 2. A leakage-safe evaluation protocol combining nested cross-validation, calibration analysis, and subject-level validation. 3. An empirical study demonstrating when deep learning does and does not outperform classical machine learning on small clinical datasets. Ultimately, the research aims to support systematic, reproducible, and clinically reliable decision support systems for multi-disease prediction.

Previous studies face several challenges that limit their applicability in real-world healthcare environments. Many models rely on complex architectures with large parameter counts, resulting in high computational cost and limited interpretability. Additionally, clinical datasets often exhibit issues such as small sample sizes, missing values, feature redundancy, and class imbalance, which can lead to overfitting and unstable predictions. Inconsistent preprocessing methods, evaluation protocols, and dataset-specific tuning across studies further hinder fair comparisons and reproducibility. These challenges highlight the need for a unified and robust deep learning framework capable of handling heterogeneous biomedical datasets effectively.

## Literature survey

In recent research [6] on Type 2 diabetes prediction indicates that traditional machine learning models such as Decision Trees, Random Forests, KNN, and Naïve Bayes exhibit limited performance on sparse and high-dimensional clinical datasets, often achieving accuracies below 85%. Although deep learning models, including CNNs, LSTMs, and autoencoder-based architectures, provide moderate improvements, their effectiveness is frequently hindered by feature redundancy, overfitting, and sensitivity to data sparsity and imbalance. Comparative studies using metrics such as accuracy, F1-score, AUC, and Hamming loss demonstrate that sparsity-aware hybrid deep learning frameworks consistently outperform standalone models. These findings highlight the need for advanced architectures that integrate effective feature selection and robust classification to improve predictive reliability in real-world diabetes datasets.

Parkinson’s disease detection using voice signals show that traditional machine learning classifiers such as KNN, Decision Tree, Random Forest, and MLP typically achieve accuracies in the range of 90–96%, but their performance is often affected by class imbalance and limited feature representation [7]. Hybrid and ensemble approaches have demonstrated improved robustness, reporting accuracies between 97% and 98.5% by combining complementary classifiers. Deep learning models, particularly CNN-based architectures, further enhance discriminative feature learning and achieve accuracies above 98%. Notably, lightweight CNN-based ensemble frameworks integrated with data balancing techniques such as SMOTE and cluster sampling have attained accuracies exceeding 99%, along with superior F1-score and AUC values, highlighting their effectiveness for reliable early Parkinson’s disease diagnosis. Despite the high accuracies reported by these models, many studies rely on recording-level splits or heavily engineered datasets, which may inflate performance and limit generalization to real-world clinical settings.

Parkinson’s disease prediction using handwritten images reveal a gradual shift from conventional machine learning methods to deep learning–based models [8]. Traditional classifiers such as SVM, KNN, and Random Forest typically achieve accuracies between 74% and 92%, limited by handcrafted features and small datasets. In contrast, CNN and transfer learning approaches using architectures like VGG, Dense Net, and ResNet report improved accuracies in the range of 95%–98.45%. Recent hybrid and ensemble-based deep learning frameworks further enhance performance, achieving accuracies above 99%, thereby demonstrating the effectiveness of fusion strategies for reliable early Parkinson’s disease diagnosis.

Zhuang et al. [9] reported that conventional machine learning classifiers, including LR, SVM, Random Forest, and boosting-based methods, achieve only moderate performance, with accuracies typically below 78% and reduced F1-scores and recall under class-imbalanced conditions. Although advanced ensemble models such as LightGBM and CatBoost offer marginal improvements, their sensitivity remains limited on small- and medium-scale datasets. In contrast, hybrid frameworks that integrate CNN-based deep feature extraction with soft-voting ensemble strategies demonstrate substantial and statistically significant gains, achieving accuracies above 85% on expanded datasets and up to 98% on large, high-dimensional datasets, with F1-score and recall exceeding 0.98. Paired statistical tests further confirm the robustness of these improvements (p < 0.01 or p < 0.05), highlighting the superiority of hybrid CNN–ensemble models in terms of discrimination capability, stability, and generalization for medical diagnosis applications.

Naeem et al. [10] highlighted the effectiveness of voice-based biomarkers combined with machine learning for early Parkinson’s disease detection, where conventional classifiers typically achieve accuracies of 75–85%, while advanced models such as SVM and Random Forest report improved performance in the range of 90–96%. Ensemble and hybrid approaches further enhance reliability, yielding accuracy gains of approximately 5–7% and F1-scores above 0.94. Moreover, the integration of class balancing and feature selection techniques such as SMOTE and PCA has been shown to boost performance, with some frameworks achieving accuracies up to 97–100% and near-perfect recall. These statistical comparisons confirm the robustness and clinical potential of voice-based intelligent diagnostic systems for PD.

Regarding heart disease prediction, Tougui et al. [11] reported that traditional machine learning classifiers such as Logistic Regression, Naïve Bayes, and SVM generally achieve accuracies between 82% and 84% on benchmark datasets, with performance varying across data mining platforms. Advanced models, particularly Artificial Neural Networks, demonstrate superior results, attaining accuracies up to 85.86% with sensitivity above 83%, while SVM-based models exhibit high specificity reaching 94.38%. Moreover, ensemble and hybrid approaches further improve prediction performance by approximately 5–8% over individual classifiers. These statistical findings emphasize the influence of both algorithm selection and computational platform on diagnostic reliability.

Deberneh and Kim [12] indicated that machine learning–based Type 2 Diabetes prediction models achieve moderate performance, with reported accuracies and AUC values typically ranging from 70% to 78%, depending on data sources and model design. Early screening approaches using national survey datasets and single classifiers such as SVM or logistic regression showed limited accuracy, particularly for prediabetes detection. More recent studies employing electronic health records and ensemble learning strategies demonstrated improved robustness, with cross-validation accuracies reaching nearly 80% when multi-year longitudinal data were incorporated. Furthermore, extending traditional predictors with biochemical and lifestyle features consistently yielded measurable accuracy gains, highlighting the importance of data-driven feature selection and temporal health information in T2D risk prediction as shown in Table 1.

**Table 1:** Summary of literature on disease prediction models.

Author(s) & Year	Disease Focus	Dataset Used	Model Used	Key Performance Metrics
In 2025, Shams et al. [13]	Diabetes	PIMA Indian Diabetes Dataset (768 samples)	RFE–GRU hybrid model with feature selection	Accuracy: 90.70%, Precision: 90.50%, Recall: 90.70%, F1-score: 90.50%, AUC: 0.9278
In 2025, Abdullah [14]	Heart Disease	CDC Cardiac Disease Dataset (~300,000 samples)	Enhanced Multilayer Perceptron (EMLP)	Accuracy: 92.00%, improved precision, recall, and F1-score over RF, SVM, KNN
In 2025, Valarmathi et. al [15]	Parkinson’s Disease	Voice-based PD dataset	Autoencoder-based Feature Extraction + DNN (FB-DNN)	Accuracy: 96.15%, superior to conventional ML classifiers
In 2025, Bataineh & Manacek [16]	Heart Disease	Cleveland Heart Disease Dataset	Hybrid MLP with Particle Swarm Optimization (MLP–PSO)	Accuracy: 84.61%, outperforming DT, RF, SVM, KNN
In 2025, Yasmeen et al [17]	Heart Disease	Kaggle & UCI CVD datasets	TabNet + XGBoost stacked ensemble	Improved Accuracy, F1-score, ROC-AUC, MCC over baseline ML and DL models
In 2024, Rahman et al. [18]	Heart Disease	UCI Cleveland Dataset	Self-attention–based Transformer model	Accuracy: 96.51%, highest among compared state-of-the-art approaches

Despite notable performance improvements reported in recent studies, several critical research gaps remain. Most existing models rely on disease-specific and curated datasets, limiting their generalizability across heterogeneous clinical environments and multi-disease scenarios. High-performing deep and transformer-based models often suffer from increased computational complexity and limited interpretability, restricting real-time deployment in resource-constrained healthcare settings. Additionally, many approaches emphasize accuracy-centric evaluation while overlooking robustness, scalability, and dynamic feature adaptation in longitudinal patient data. These limitations indicate the need for a unified, lightweight, and interpretable predictive framework that balances accuracy, generalization, and practical clinical applicability.

## Research design & methodology

**Fig 1:**
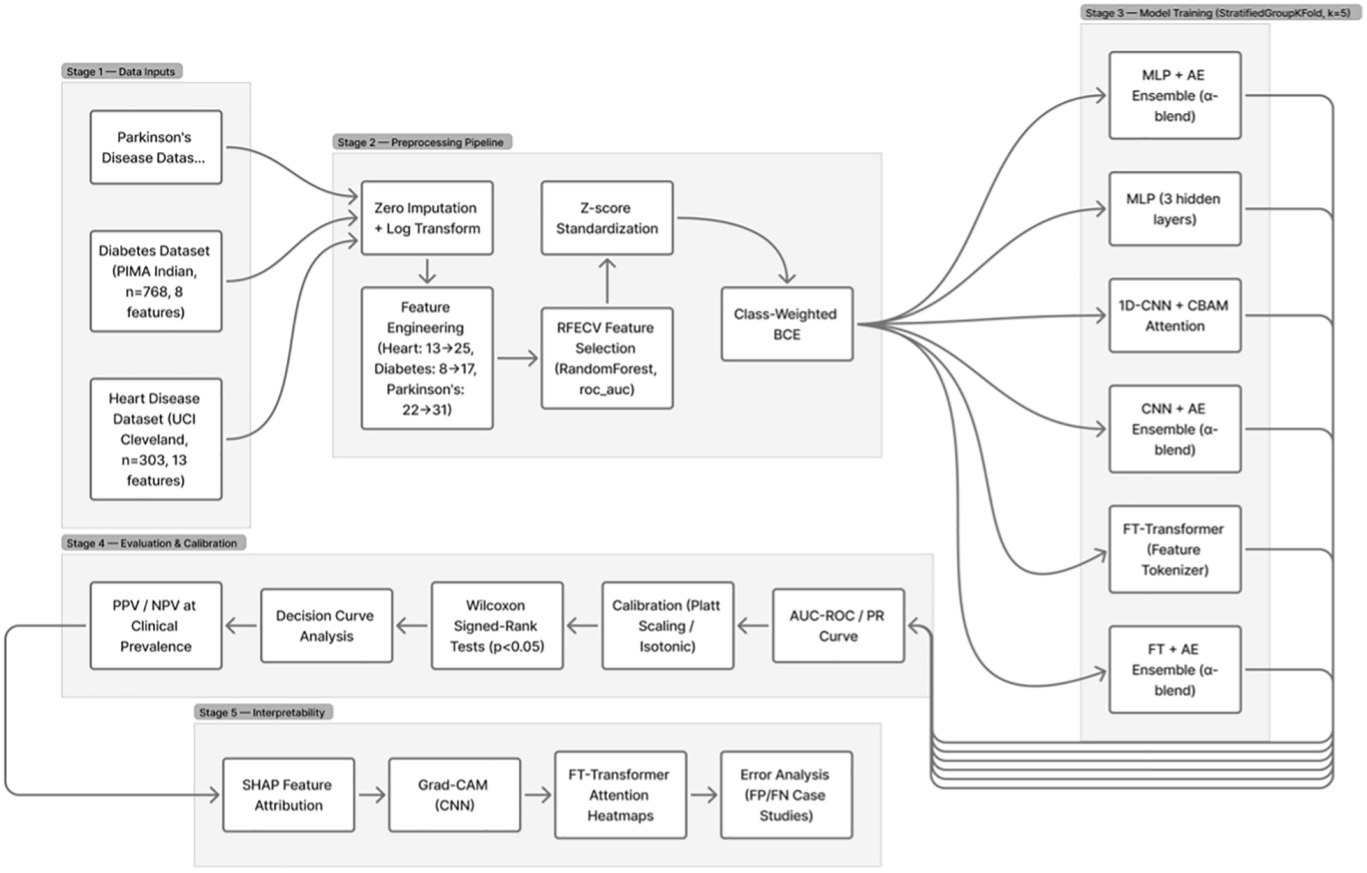
Multi-model disease prediction framework.

This section presents the design of a unified deep learning framework for disease prediction across multiple clinical datasets. The framework follows a modular architecture that enables dataset-aware instantiation while maintaining a consistent underlying methodology as shown in Fig 1.

We propose a unified deep learning framework for binary disease classification that systematically addresses the challenge of heterogeneous clinical datasets. Rather than developing isolated solutions for each prediction task, we introduce a cohesive system that adapts its architectural components based on dataset-specific characteristics while preserving a consistent methodological foundation.

The framework operates on the following key principle: different clinical datasets exhibit distinct properties varying feature dimensionalities, feature interaction patterns, data distributions, and modality types that benefit from tailored architectural choices. By establishing a common training protocol, evaluation methodology, and modular component library, the framework enables fair comparison across architectures while allowing each dataset to leverage the most appropriate model instantiation.

The high-level pipeline follows a standard supervised learning paradigm: raw clinical data undergoes domain-aware preprocessing, passes through standardization and feature representation, then through selected model architecture from our model library, and finally produces disease probability predictions via sigmoid activation. The key contribution lies in the systematic approach to model selection and the principled justification for dataset-specific architectural decisions.

We note that the individual model architectures employed (MLP, CNN, FT-Transformer, Autoencoder) are established methods from the deep learning literature. The contribution of this work is not in proposing novel architectures, but in their systematic integration within a unified benchmarking framework encompassing dataset-aware preprocessing, principled evaluation via nested cross-validation, comprehensive interpretability analysis, and rigorous statistical comparison against classical baselines [19].

Overview of the proposed multi-model disease prediction framework. The pipeline comprises five sequential stages: (1) three independent tabular clinical datasets (Heart Disease, n = 303; Diabetes, n = 768; Parkinson’s Disease, n = 195), each processed independently through the remainder of the pipeline; (2) a unified preprocessing pipeline including zero imputation, log transformation of skewed features, dataset-specific feature engineering (Heart: 13 → 25, Diabetes: 8 → 17, Parkinson’s: 22 → 31 features), RFECV feature selection (Random Forest base estimator, roc_auc criterion), z-score standardization, and class-weighted binary cross-entropy to handle class imbalance note that SMOTEENN oversampling was disabled (--no-augmentation) in the final experimental runs; (3) parallel training of six model configurations under StratifiedGroupKFold cross-validation (k = 5 outer, k = 3 inner folds), with subject-level splitting enforced for Parkinson’s disease: three standalone models (MLP, 1D-CNN+CBAM, FT-Transformer) and three Autoencoder ensembles (MLP + AE, CNN + AE, FT + AE, all with α-blending) all trained with Cosine Annealing with Warm Restarts scheduler, Stochastic Weight Averaging (SWA), label smoothing (ε = 0.05), and Test-Time Augmentation (TTA, n = 10); (4) comprehensive evaluation including AUC-ROC, Platt Scaling/ Isotonic calibration, Wilcoxon signed-rank significance testing (p < 0.05), decision curve analysis, and clinical PPV/NPV at realistic disease prevalence; and (5) multi-method interpretability via SHAP feature attribution, Grad-CAM (CNN), FT-Transformer cross-attention heatmaps, and false positive/negative case studies as shown in Fig 1.

### Unified deep learning framework design

#### Model modularity.

The framework establishes several components that remain consistent across all dataset instantiations, ensuring methodological coherence and experimental reproducibility [20]. At its core, the framework adopts a modular design philosophy where individual components feature encoders, attention mechanisms, classification heads, and auxiliary networks can be composed in various configurations. This modularity enables systematic exploration of architectural choices while maintaining a unified codebase and evaluation protocol. The individual model architectures (MLP, CNN, Transformer, Autoencoder) are not proposed as novel designs; the contribution lies in their principled integration and dataset-aware selection within a unified framework. The framework implements five model families: Multi-Layer Perceptron (MLP), 1D Convolutional Neural Network (CNN), Feature-Tokenizer Transformer (FT-Transformer), Autoencoder-based classifiers, and Ensemble models that combine primary classifiers with autoencoder representations.

#### Training protocol.

A consistent training protocol governs all experiments using gradient-based optimization with weight decay regularization. Learning rate scheduling employs one of three strategies selected via hyperparameter tuning: ReduceLROnPlateau, StepLR, or Cosine Annealing with Warm Restarts. Patience-based early stopping (monitoring validation loss) prevents overtraining. Batch processing uses dataset-appropriate batch sizes with incomplete final batches dropped to ensure normalization layer stability.

#### Regularization.

The framework incorporates multiple regularization techniques to prevent overfitting and improve generalization. Dropout is applied with progressive rates across layers, with higher rates in early layers and reduced rates in later layers. Batch normalization is applied after linear and convolutional layers in early stages to stabilize training. Weight decay provides L2 regularization uniformly across all model families. For imbalanced datasets, class weighting with inverse frequency weighting is integrated into the binary cross-entropy loss function.

### Feature representation and input handling

The preprocessing pipeline transforms raw clinical data into model-ready representations through the following stages:

#### Standardization and cleaning.

All features undergo z-score standardization. Dataset-specific cleaning includes imputation of biologically impossible zeros with median values, log transformation of skewed features, and removal of identifier columns.

#### Feature engineering.

Detailed per-dataset derived features are documented below. These engineered features were derived directly from established clinical risk relationships reported in prior cardiovascular, metabolic, and acoustic analysis studies, ensuring domain relevance. Dataset-specific feature engineering is applied after cleaning and prior to feature selection, expanding each dataset’s raw feature space with domain-motivated interaction terms, clinical ratio features, polynomial terms, and threshold-based binary indicators. All derived features are computed within each training fold exclusively, with the same transformations applied (without re-fitting) to the validation fold to prevent leakage.

Heart Disease (13 → 25 features): Twelve features are derived: four pairwise interaction terms capturing clinically coupled variables (exang×oldpeak exercise angina and ST depression both reflect ischemic burden; cp×thalach symptom–cardiac output coupling; age×thalach; chol×age); two ratio features (hr_reserve_ratio = ``thalach``/(220 − age) encoding the proportion of age-predicted maximum heart rate achieved; bp_age_ratio`` = ``trestbps``/age-adjusted-normal); two polynomial terms (oldpeak², thalach²) capturing non-linear risk thresholds; one composite risk score (sum of binary risk flags: sex, exang, fbs); two clinical threshold bins (chol_risk_bin: 0/1/2 for <200/ > 200/ > 240 mg/dL; age_bucket: < 45/45–60/ > 60); and one ischemic burden ratio (oldpeak``/``thalach).

Diabetes (8 → 17 features): Nine features are derived: three interaction terms (BMI × Age; Glucose×Insulin; Glucose×BMI); one normalised ratio (Pregnancies/Age); the validated clinical biomarker HOMA-IR = (Glucose × Insulin)/ 405, which approximates insulin resistance; a four-class ordinal BMI category variable following WHO cut-points (<18.5 underweight, 18.5–25 normal, 25–30 overweight, > 30 obese); a binary high-pregnancy flag (≥6 pregnancies); and two non-linear terms (Glucose²; BMI×Glucose``/1000 as a composite insulin resistance index).

Parkinson’s Disease (22 → 31 features): Nine aggregate and ratio statistics are derived across the five jitter measures (MDVP:Jitter(%), MDVP:Jitter(Abs), MDVP:RAP, MDVP:PPQ, Jitter:DDP) and six shimmer measures (MDVP:Shimmer, MDVP:Shimmer(dB), Shimmer:APQ3, Shimmer:APQ5, MDVP:APQ, Shimmer:DDA): mean-product interaction (jitter_mean`` × ``shimmer_mean), aggregate sum (jitter_total, shimmer_total), cross-measure standard deviation (jitter_std, shimmer_std), range (jitter_range, shimmer_range), ratio (jitter_mean``/ ``shimmer_mean) reflecting relative vocal perturbation, and a harmonicity–frequency interaction (HNR × spread1).

#### Feature selection via recursive feature elimination (RFE).

Given an input feature matrix $X\in R^{n\times d}$ with n samples and d features, and target vector $y\in \{\text{0},\text{1}{\}}^{n}$, RFE iteratively identifies the optimal feature subset $S^{*}\subseteq \{\text{1},\ldots ,d\}$ through the following procedure:

1. Train a base estimator h on (X,y) and compute feature importance scores $w_{j}$ forj=1,…,d
2. Eliminate features with lowest importance:$S_{t+\text{1}}=S_{t}\backslash \{\text{arg}{\text{min}}_{j\in S_{t}}w_{j}\}$
3. Repeat until $|S_{t}|=k$, where k is determined by cross-validated AUC maximization

The base estimator for RFECV is a RandomForestClassifier (100 estimators, max_depth = 5, min_samples_split = 5) using Gini importance. The CV scoring function is roc_auc, with step = 1 (one feature eliminated per iteration). Dataset-specific minimum feature thresholds are enforced: Heart Disease ≥ 5, Diabetes ≥ 3, Parkinson’s ≥ 8.

The optimal feature count $k^{*}$ is selected as:


$$k^{*}=\text{arg}\underset{k}{\text{max}}\text{CV}-\text{AUC}(h,X_{S_{k}},y)$$ (1)

#### Fold safety.

All preprocessing, feature engineering, feature selection, imputation, standardization, and quantile transformation steps are fit exclusively on the training fold and applied (transform-only) to the validation/test fold within each cross-validation iteration to prevent any form of data leakage.

Input shape conventions differ by architecture: tabular models (MLP, FT-Transformer, Autoencoder) receive input tensors of shape $\left(n,d^{\prime }\right)$ where $d^{\prime }=|S^{*}|$ represents the selected feature count as determined by Equation (1), while convolutional models (CNN) receive tensors of shape $\left(n,\text{1},d^{\prime }\right)$ with an added channel dimension.

### Model families within the framework

**Fig 2:**
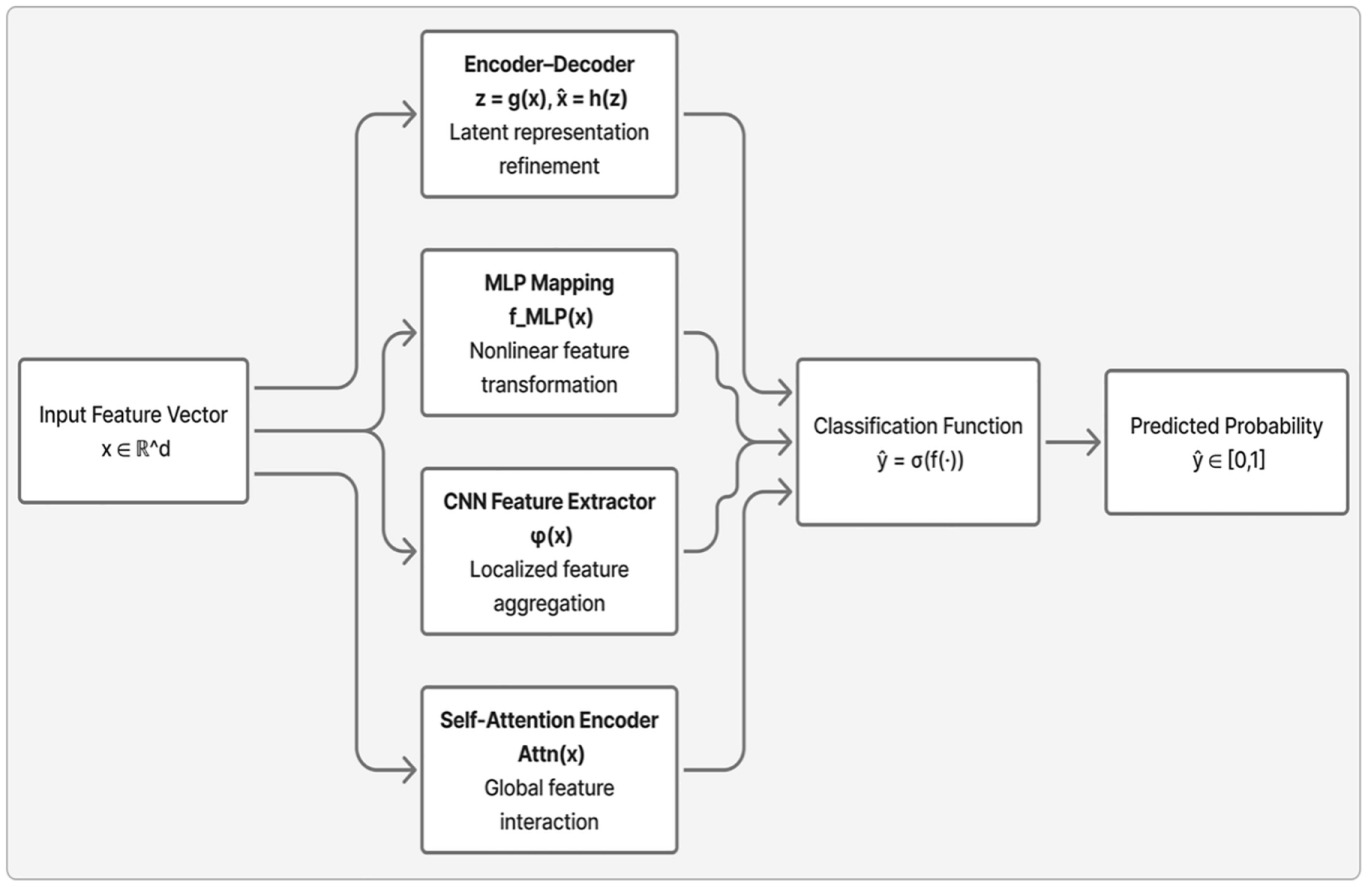
Abstract representation of the model families considered in this study.

To clarify the distinctive mechanisms by which each architecture processes clinical features, Fig 2. provides an abstract representation of the model families employed in the proposed framework. While all models share a unified training objective and classification head, they differ fundamentally in their feature transformation strategies:

MLPs apply non-linear mappings to global feature vectors, CNNs exploit local adjacency in ordered inputs, Transformers model global pairwise interactions via self-attention, and Autoencoder-augmented ensembles integrate unsupervised latent refinements.

#### Multi-layer perceptron (MLP).

The Multi-Layer Perceptron serves as the foundational architecture within the framework, providing a strong baseline for tabular clinical data classification in 2021, Gorishniy et al [21]. The MLP implements a composition of nonlinear mappings through successive hidden layers:


$$f_{MLP}(x)=\sigma (W_{L}\cdot \phi (W_{L-\text{1}}\cdot \phi (\ldots \phi (W_{\text{1}}\cdot x+b_{\text{1}})\ldots )+b_{L-\text{1}})+b_{L})$$ (2)

Where ϕ(·) denotes the activation function (GELU for smoother gradients or ReLU for computational efficiency), σ(·)  is the sigmoid output producing probability estimates, and each layer incorporates batch normalization for training stability and dropout for regularization. The MLP architecture is particularly suitable for structured clinical data where complex feature interactions are diagnostically important, but no inherent spatial or sequential structure exists among features. Unlike decision tree ensembles, MLPs can learn smooth decision boundaries and capture nonlinear interactions between continuous clinical measurements, making them effective for datasets with predominantly numerical features such as metabolic markers or physiological measurements.

#### 1D convolutional neural network (CNN).

The CNN extends the framework to exploit local structure among ordered feature sequences. While CNNs are traditionally applied to image and signal data, they can be effectively adapted for tabular clinical data when features exhibit meaningful adjacency relationships for example, acoustic measurements grouped by type (jitter variants, shimmer variants) or physiological readings ordered by measurement time [22]. The CNN architecture decomposes into feature extraction and classification stages, as formalized in Equation (3):


$$f_{CNN}(x)=\sigma (g_{cls}(g_{extract}(x)))$$ (3)

Where $g_{extract}:R^{d}\to R^{d^{\prime }}$ applies learnable convolutional filters across the feature dimension to extract local patterns, optionally enhanced by the Convolutional Block Attention Module (CBAM) for channel and spatial attention refinement. The classification head $g_{cls}$ maps the extracted feature representations to class probabilities. Residual connections are incorporated for deeper configurations to facilitate gradient flow. This architecture is particularly suited for clinical datasets where related measurements are positioned adjacently in the feature vector, allowing convolutional kernels to learn diagnostically meaningful patterns spanning multiple related features [23].

#### Feature-tokenize transformer (FT-transformer).

The FT-Transformer represents a recent architectural advancement specifically designed for tabular data, adapting the attention mechanism originally developed for natural language processing to structured clinical records. Unlike MLPs that process all features simultaneously through dense layers, or CNNs that assume local feature relationships, the FT-Transformer treats each feature as an individual token and learns arbitrary pairwise and higher-order interactions through self-attention [24]. The architecture learns global feature interactions as defined in Equation (4):


$$f_{FT}(x)=\sigma ({\text{MLP}}_{cls}(\text{Transformer}([e_{cls};E\cdot x])))$$ (4)

Where $E\in R^{d_{token}\times d}$ is a learnable embedding matrix that projects each scalar feature into a $d_{token}$-dimensional token representation, $e_{cls}$ is a learnable classification token appended to the sequence, and the Transformer backbone applies multi-head self-attention over the complete token sequence. Following [21], this architecture captures global feature dependencies without imposing locality assumptions, making it particularly effective for clinical datasets where distant features may have strong diagnostic interactions (e.g., age interacting with cholesterol levels in cardiovascular risk assessment). The attention weights additionally provide interpretability by revealing which feature pairs the model considers most relevant for prediction.

#### Autoencoder-based models.

The auto encoder architecture serves a complementary role within the framework, providing unsupervised representation learning that captures the underlying structure of clinical feature distributions [25]. By learning to compress and reconstruct input features, the autoencoder discovers latent representations that encode meaningful relationships among clinical measurements such as metabolic correlations between glucose, insulin, and BMI in diabetes data, or acoustic feature relationships in Parkinson’s voice recordings. The autoencoder learns a compressed latent representation by minimizing reconstruction error (Equations 5 and 6):


$$z=f_{enc}(x),\;\hat{x}=g_{dec}(z)$$ (5)

$$L_{AE}=∥x-\hat{x}\overset{\text{2}}{\underset{\text{2}}{∥}}$$ (6)

Where $f_{enc}:R^{d}\to R^{k}$ is the encoder mapping inputs to a lower-dimensional latent space (k<d), and $g_{dec}:R^{k}\to R^{d}$ is the decoder reconstructing the original features. Three variants are implemented within the framework: (1) Standard Autoencoder for purely unsupervised representation learning, where the encoder is frozen after training and used as a feature extractor; (2) Supervised Autoencoder with joint loss $L=\alpha L_{AE}+(\text{1}-\alpha )L_{cls}$ that simultaneously optimizes reconstruction and classification objectives, encouraging discriminative latent representations; and (3) Autoencoder Classifier with a pre-trained encoder whose weights are fine-tuned for classification. Conservative compression ratios are employed to preserve sufficient discriminative information while providing noise-robust representations that generalize better than raw features.

#### Ensemble strategy.

The ensemble strategy represents the most sophisticated configuration within the framework. The core insight is that primary classifiers (MLP, CNN, or FT-Transformer) and autoencoders capture complementary information: the primary classifier optimizes directly for the classification boundary, while the autoencoder learns representations that capture the underlying data distribution regardless of class labels [26]. The ensemble combines predictions from both pathways through weighted averaging, as shown in Equation (7):


$$\hat{y}=\alpha \cdot p_{ae}+(\text{1}-\alpha )\cdot p_{primary}$$ (7)

Where $p_{ae}$ is the prediction from the autoencoder-based classifier, $p_{primary}$ is the prediction from the primary architecture (MLP, CNN, or FT-Transformer), and α∈[0,1] controls the relative contribution of each component. The ensemble weight α is initialized based on component validation performance during cross-validation, with higher weight assigned to the better-performing component. This fusion is particularly effective for imbalanced datasets, where the autoencoder’s representations learned from reconstruction objectives applied to all samples provide regularization against overfitting to the majority class.

#### Attention mechanisms.

Attention mechanisms serve as reusable enhancement modules within the unified framework, selectively activated based on dataset characteristics to improve feature representation.

#### CBAM.

Applies sequential channel and spatial attention to refine CNN feature representations, helping the model focus on clinically relevant feature combinations.

#### self-attention in transformers.

The FT-Transformer utilizes multi-head self-attention defined in Equation (8):


$$Attention(Q,K,V)=softmax(\frac{QK^{T}}{\sqrt{d_{k}}})V$$ (8)

This enables dynamic feature interaction modeling, valuable for medical data where symptom combinations may have non-additive effects.

### dataset-aware framework instantiation

This section explains the rationale for architectural choices on each dataset. The framework is instantiated differently based on each dataset’s unique characteristics using the following clear set of dataset-aware formal rules:

Small sample size + high clinical interaction complexity - > FT-Transformer: For structured datasets where global feature interactions (e.g., age × cholesterol × blood pressure) are non-linear but data points are moderate, self-attention architectures capture the complex dependencies.

Physiologically linked sub-groups + Moderate sample size - > Ensemble (MLP/CNN + AE): For metabolic datasets containing highly correlated subsets (e.g., glucose, insulin, BMI), combining discriminative layers with autoencoder-derived latent representations optimizes generalization.

Adjacency structures in feature vectors + Severe data scarcity - > Regularized MLP/CNN: For voice/acoustic datasets with grouped correlated metrics (e.g., jitter variants, shimmer variants) and severe class imbalance, highly regularized MLPs or CNNs with large kernels are prioritized, as complex attention mechanisms rapidly overfit tiny sample sizes.

#### Instantiation for heart disease dataset.

The UCI Heart Disease dataset is a widely studied benchmark for cardiovascular risk prediction, comprising 303 patient records from the Cleveland Clinic. Each record contains 13 clinical features that represent established cardiac risk factors: demographic variables (age, sex), physiological measurements (resting blood pressure, serum cholesterol, maximum heart rate achieved), diagnostic indicators (chest pain type, resting ECG results, exercise-induced angina, ST depression), and categorical markers (fasting blood sugar, number of major vessels colored by fluoroscopy, thalassemia status). The class distribution is approximately balanced, with patients classified as having or not having significant coronary artery disease.

The dataset exhibits common clinical data quality issues that require careful preprocessing. Invalid thalassemia values (thal = 0), which represent missing or unrecorded entries, are replaced with the mode to preserve categorical integrity. Physiological outliers in resting blood pressure (trestbps), serum cholesterol (chol), ST depression (oldpeak), and number of major vessels (ca) are capped using IQR winsorization to reduce the influence of extreme values while retaining clinical plausibility. The architectural specificities for the heart disease dataset are detailed in Table 2.

**Table 2:** Model configuration for heart disease dataset.

Model	Architecture Summary
MLP	64-32-16 hidden, GELU activation
CNN	2 × Conv32 + CBAM + MaxPool
FT-Transformer	2 layers, 2 heads, token_dim = 32

The moderate feature dimensionality (13 features) combined with the limited sample size (303 patients) necessitates compact architectures to prevent overfitting. The clinical nature of heart disease diagnosis involves complex interactions among risk factors—for example, the combined effect of age, cholesterol, and blood pressure on cardiovascular risk and ischemic burden is nonlinear, synergistic, and multiplicative. This motivates architectures capable of learning global feature interactions.

*Proposed Model for Heart Disease: FT + AE Ensemble:* The FT + AE Ensemble is selected for heart disease as it combines the FT-Transformer’s self-attention mechanism with an autoencoder’s latent representation learning. The self-attention mechanism can capture arbitrary pairwise and higher-order interactions among all 13 cardiac risk factors without assuming spatial locality, which aligns with clinical reasoning about heart disease involving multiple interacting factors (e.g., age × cholesterol × blood pressure). The integration of the autoencoder further stabilizing the representations, mitigating the high variance often seen in transformer models on limited training samples, ultimately achieving the highest AUC and robust recall.

#### Instantiation for diabetes dataset.

The PIMA Indians Diabetes dataset originates from a longitudinal study conducted by the NIDDK, focusing on diabetes incidence among Pima Indian women aged 21 years or older. The dataset contains 768 patient records with 8 clinical measurements that serve as metabolic and physiological markers: pregnancies count, plasma glucose concentration (2-hour oral glucose tolerance test), diastolic blood pressure, triceps skin fold thickness, 2-hour serum insulin, body mass index (BMI), diabetes pedigree function (a genetic risk score), and age. The class distribution is imbalanced with 500 negative cases (65.1%) and 268 positive cases (34.9%), reflecting the natural prevalence of diabetes in the study population and presenting a classification challenge that requires careful handling.

The dataset exhibits a known issue where missing values are encoded as zeros in physiologically impossible contexts for instance, zero values for glucose, blood pressure, skin thickness, insulin, or BMI are biologically implausible. These entries are replaced with column-wise medians computed within each cross-validation training fold to prevent data leakage. Additionally, the Insulin and DiabetesPedigreeFunction features exhibit right-skewed distributions spanning multiple orders of magnitude; log transformation is applied to these features to reduce skewness and improve model convergence.

The larger sample size (768 patients) relative to the heart disease dataset permits wider hidden layers (in Table 3) without excessive overfitting risk. Higher dropout rates are employed to counteract the class imbalance, and class-weighted loss functions ensure the minority positive class receives appropriate training emphasis. The metabolic features exhibit underlying biological correlations specifically, glucose levels, baseline insulin production, and high BMI are physiologically interlocked through systemic insulin resistance and beta-cell dysfunction pathways strongly motivating representation learning approaches that can capture these exact latent relationships.

**Table 3:** Model configuration for diabetes disease dataset.

Model	Architecture Summary
MLP	128-64-32 hidden, GELU activation
CNN	2 × Conv64 + CBAM + GAP
FT-Transformer	2 layers, 4 heads, token_dim = 64

*Proposed Model for Diabetes: CNN+AE Ensemble:* The CNN+AE Ensemble is selected for the Diabetes dataset because convolutional layers capture structured relationships among metabolically linked features such as glucose, insulin, and BMI, while the autoencoder learns latent representations that improve generalization under class imbalance.

#### Instantiation for Parkinson’s disease dataset.

The Parkinson’s disease dataset originates from voice recording analysis studies at the University of Oxford, comprising 195 voice recordings from 32 individuals (23 with Parkinson’s disease, 8 healthy controls, with one subject contributing recordings under both conditions). Each recording is characterized by 22 acoustic features extracted from sustained vowel phonations, capturing various dimensions of vocal impairment associated with Parkinson’s disease. These features include fundamental frequency variation measures (jitter variants: absolute, relative, RAP, PPQ, DDP), amplitude variation measures (shimmer variants: local, dB, APQ3, APQ5, APQ11, DDA), harmonicity measures (NHR, HNR), and nonlinear dynamics measures (RPDE, DFA, spread1, spread2, D2, PPE). The class distribution is highly imbalanced with 147 positive cases (75.4%) and 48 negative cases (24.6%), reflecting the study design where multiple recordings were collected from each Parkinson’s patient.

The preprocessing pipeline addresses the unique characteristics of acoustic feature data. Critically, subject IDs are extracted from the recording name column using regex pattern matching (e.g., `phon_R01_S01_1` → `S01`), mapping all 195 recordings to their 32 underlying subjects. Cross-validation for the Parkinson’s dataset exclusively uses `StratifiedGroupKFold` (outer loop) and `StratifiedGroupKFold` (inner loop) with subject-level grouping, strictly guaranteeing that all recordings from any given subject are isolated entirely within either the training or the testing fold achieving zero subject leakage. A Leave-One-Subject-Out (LOSO) cross-validation with 32 folds was also conducted to provide the most rigorous possible evaluation of subject-level generalization. Standard scaling (z-score normalization) is applied uniformly to all 22 acoustic features to ensure comparable feature magnitudes, as the raw features span different numerical ranges (e.g., frequency measurements in Hz versus ratio measurements).

The high feature dimensionality (22 features) relative to the limited sample size (195 recordings) creates a challenging learning scenario that requires careful regularization to prevent overfitting. Importantly, the acoustic features exhibit natural physiological groupings reflecting underlying motor impairment jitter variants measure glottal frequency perturbation, shimmer variants capture amplitude perturbation indicative of breathiness, and nonlinear dynamics features isolate voice signal complexity. These related measurements are often positioned adjacently in the feature vector, creating local correlations that convolutional architectures can exploit. The large initial kernel size (k=15, in Table 4) is specifically chosen to capture patterns spanning multiple related acoustic measures within each feature group.

**Table 4:** Model configuration for Parkinson’s disease dataset.

Model	Architecture Summary
MLP	256-128-64 hidden, ReLU activation
CNN	Conv64(k = 15) + 3 × ResBlock128 + CBAM + Conv256
FT-Transformer	5 layers, 4 heads, token_dim = 64

The severe class imbalance (75% positive) requires targeted mitigation strategies. AUC serves as the primary evaluation metric because of its threshold independence and insensitivity to class distribution. Autoencoder-derived representations provide features that are learned from reconstruction objectives rather than class labels, making them inherently robust to class frequency biases. Class-weighted loss functions ensure the minority negative class (healthy controls) receives proportionally higher training emphasis. Data augmentation techniques (e.g., SMOTEENN) were deliberately disabled for the final experiments because the small negative class (48 samples) provides insufficient diversity for synthetic sample generation without introducing artificial patterns; the ablation study confirms this decision.

*Proposed Model for Parkinson’s: Multi-Layer Perceptron (MLP):* The standalone MLP architecture is selected for the Parkinson’s dataset, as it achieved the highest discriminative performance (AUC 0.754) among all tested deep learning models in this highly constrained data regime. The severe sample size limitations (only 32 subjects total, evaluated via strict LOSO cross-validation) and severe class imbalance make training deep networks exceedingly difficult. In this context, simpler architectures with fewer parameter dependencies generalize better. The MLP effectively learns non-linear decision boundaries within the 22-dimensional acoustic feature space without the overfitting risks associated with more complex representation learning or attention mechanisms on such a small cohort.

## Implementation detail

This section details the complete experimental configuration used for all reported results, ensuring full reproducibility.

### Training configuration

Table 5a and 5b summarize the training parameters for standalone and ensemble model families, respectively.

**Table 5:** (a) Standalone model training configuration (MLP, CNN, FT-transformer). (b) Ensemble model training configuration (MLP + AE, CNN + AE, FT + AE).

Parameter	Value
**(a) Ensemble Model Training Configuration (MLP + AE, CNN + AE, FT + AE)**	
Maximum Epochs	40
Optimizer	Adam (β₁ = 0.9, β₂ = 0.999)
LR Scheduler	Cosine Annealing with Warm Restarts (T₀ = 10, T_mult = 2, η_min = 1e-6)
Early Stopping	Monitors validation loss, patience tuned per dataset (10–20 epochs), min_delta = 1e-4
Label Smoothing	ε = 0.05
Stochastic Weight Averaging (SWA)	Enabled; SWA LR = 1e-4; starts at 75% of training
Test-Time Augmentation (TTA)	10 copies with Gaussian noise, predictions averaged
Probability Calibration	Isotonic regression (Platt scaling) on validation fold
Feature Selection (RFECV)	Enabled by default
HP Search	Optuna TPE, 15 trials per inner fold
Random Seed	42
Incomplete Batches	Dropped (drop_last = True) for Batch Norm stability
**(b) Ensemble Model Training Configuration (MLP + AE, CNN + AE, FT + AE)**	
Epochs (4-stage)	AE: 100, AE-Classifier: 50, MLP: 50, Ensemble fine-tuning: 20
Optimizer	Adam
Stochastic Weight Averaging (SWA)	Enabled
Probability Calibration	Platt scaling on validation fold
HP Search	Grid search with random subsampling, 6 trials
Feature Selection (RFECV)	Enabled by default
Random Seed	42

### Hyperparameter search spaces

Hyperparameter optimization uses Optuna Bayesian search (TPE sampler, 15 trials per inner fold). The default search space is applied for all three standalone model families. The same search space structure is shared across MLP, CNN, and FT-Transformer as shown in Table 6.

**Table 6:** Standalone model search spaces.

Parameter	Heart Disease	Diabetes	Parkinson’s
Learning Rate	{5 × 10 ⁻ ⁴, 1 × 10 ⁻ ³, 2 × 10 ⁻ ³}	{5 × 10 ⁻ ⁴, 1 × 10 ⁻ ³, 2 × 10 ⁻ ³}	{1 × 10 ⁻ ³, 2 × 10 ⁻ ³}
Batch Size	{16, 32, 64}	{32, 64, 128}	{16, 32}
L2 Regularization	{1 × 10 ⁻ ⁴, 1 × 10 ⁻ ³, 1 × 10 ⁻ ²}	{1 × 10 ⁻ ⁴, 1 × 10 ⁻ ³, 1 × 10 ⁻ ²}	{1 × 10 ⁻ ⁴, 1 × 10 ⁻ ³}
Early Stopping Patience	{10, 15, 20}	{10, 15, 20}	{15, 20}
Optimizer	Adam	Adam	Adam

Ensemble models (MLP + AE, CNN + AE, FT + AE) use a separate grid search with random subsampling (6 trials) described in Table 7 and dataset wise dimension configuration shown in Table 8:

**Table 7:** Ensemble model hyperparameter grid.

Parameter	Search Space
AE Learning Rate	{1 × 10 ⁻ ³, 5 × 10 ⁻ ⁴}
MLP Learning Rate	{1 × 10 ⁻ ³, 2 × 10 ⁻ ³}
Batch Size	{16, 32}
L2 Regularization	{1 × 10 ⁻ ⁴, 1 × 10 ⁻ ³}
Freeze Encoder	{True, False}
Ensemble Weight (α)	Initialized from component validation accuracy

**Table 8:** Autoencoder architecture configuration.

Dataset	Input Dim	Latent Dim	Hidden Dims	Classifier Hidden
Heart Disease	13	8	[10]	16
Diabetes	8	6	[7]	12
Parkinson’s	22	16	[20]	24

### Nested cross-validation structure

The evaluation employs nested cross-validation to provide unbiased performance estimates while avoiding information leakage from hyperparameter tuning described in Table 9.

**Table 9:** Cross-validation configuration per dataset.

Dataset	Outer Loop	Inner Loop (Standalone)	Inner Loop (Ensemble)	Splitting Strategy	Rationale
Heart Disease	5-fold StratifiedKFold	3-fold	2-fold	Standard stratification	Balanced classes (≈55/45%)
Diabetes	5-fold StratifiedKFold	3-fold	2-fold	Standard stratification	Moderate imbalance (65/35%)
Parkinson’s	5-fold StratifiedGroupKFold	3-fold	2-fold	Subject-level grouping (32 groups)	Prevents data leakage
Parkinson’s (LOSO)	32-fold LeaveOneGroupOut	2-fold (10 Optuna trials)	2-fold (6 HP trials)	Leave-one-subject-out	Maximum rigor

Workflow per outer fold: 1. Split: Outer fold partitions data into train+val and test sets (stratified, with subject grouping for Parkinson’s) 2. Preprocess: Standardization, imputation, log-transforms, and RFECV are fit on train+val only 3. Inner CV: Hyperparameters are tuned on inner folds (train/val split within train+val) 4. Retrain: Best hyperparameters are used to retrain on the full train+val set 5. Evaluate: Final model is evaluated on the held-out test fold 6. Calibrate: Post-hoc calibration is applied using the validation subset

### Optimization details

Optimizer: Adam optimizer with default momentum parameters (β₁ = 0.9, β₂ = 0.999). Weight decay (L2 regularization) is tuned per dataset as specified in Table 9. Learning Rate Scheduling: Cosine Annealing with Warm Restarts cyclically reduces the learning rate following:


$$\eta_{t}=\eta_{min}+\frac{\text{1}}{\text{2}}(\eta_{max}-\eta_{min})(\text{1}+\text{cos}(\frac{T_{cur}}{T_{i}}\pi ))$$ (9)

With T₀ = 10 epochs and T_mult = 2 (i.e., restart periods: 10, 20, 40 epochs). The minimum learning rate η_min = 1 × 10 ⁻ ⁶. During the SWA phase (final 25% of epochs), the cosine scheduler is replaced by a fixed SWA LR of 1 × 10 ⁻ ⁴. Early Stopping: Monitors validation loss with a minimum improvement delta of 1 × 10 ⁻ ⁴. Patience is tuned as a hyperparameter (10–20 epochs). Early stopping is disabled during the SWA phase to allow weight averaging to complete. Loss Function: Binary cross-entropy with per-sample class weighting inversely proportional to class frequency. Label smoothing replaces hard targets y ∈ {0,1} with smoothed targets y’ = y(1 − ε) + ε/2, where ε = 0.05. Stochastic Weight Averaging (SWA): Activates at 75% of training, averaging model weights over subsequent epochs. After training, batch normalization statistics are updated with a forward pass over the training set before evaluation. Test-Time Augmentation (TTA): At inference, 10 copies of each test sample are generated by adding Gaussian noise, and predictions are averaged for more robust probability estimates.

### Training procedure

Loss Functions: The primary training objective is binary cross-entropy with optional class weighting given by Equation (9):


$$L_{cls}=-\frac{\text{1}}{n}\sum_{i=\text{1}}^{n}\left[w_{\text{1}}\cdot y_{i}\text{log}({\hat{y}}_{i})+w_{\text{0}}\cdot (\text{1}-y_{i})\text{log}(\text{1}-{\hat{y}}_{i})\right]$$ (10)

Where ${\hat{y}}_{i}=f_{\theta }\left(x_{i}\right)\in [\text{0},\text{1}]\;$ the model output and class weights are $w_{\text{0}},w_{\text{1}}$ are set inversely proportional to class frequencies for imbalanced datasets. For autoencoder-based models, the reconstruction loss is defined as $L_{AE}=\frac{\text{1}}{n}\sum_{i=\text{1}}^{n}∥x_{i}-{\hat{x}}_{i}\overset{\text{2}}{\underset{\text{2}}{∥}}$. Supervised autoencoders optimize a combined objective:$L=\alpha L_{AE}+(\text{1}-\alpha )L_{cls}$. Label Smoothing: To improve calibration and prevent overconfident predictions, optional label smoothing (ϵ=0.05) is applied, replacing hard targets y∈{0,1} with smoothed targets y^‘ = y(1-ϵ)+ϵ/2. Data Augmentation (Available): The framework provides multiple augmentation strategies that can be activated via command-line flags: (1) SMOTEENN SMOTE combined with Edited Nearest Neighbors; (2) Gaussian noise injection; (3) Mixup augmentation; (4) Feature dropout. However, for the final experiments reported in this study, data augmentation was deliberately disabled (--no-augmentation) based on ablation results showing that augmentation did not consistently improve performance on these small tabular datasets. The ablation study (Section 4) quantifies the contribution of augmentation when enabled.

Cross-Validation Strategy: We employ nested k-fold cross-validation to obtain unbiased performance estimates while tuning hyperparameters. The outer loop uses 5-fold stratified cross-validation for performance estimation, while the inner loop uses 2–3 fold stratified cross-validation for hyperparameter selection via Optuna Bayesian optimization (TPE sampler, 15 trials per inner fold). This approach prevents information leakage from hyperparameter tuning into final performance estimates. All splits maintain class distribution through stratified sampling. For the Parkinson’s dataset, StratifiedGroupKFold with subject-level grouping is used in both outer and inner loops to prevent subject leakage. Training Enhancements: The framework incorporates several advanced training techniques whose individual contributions are quantified via ablation study: (1) Cosine Annealing with Warm Restarts cyclically decays the learning rate following a cosine schedule, enabling escape from local minima; (2) Stochastic Weight Averaging (SWA) averages model weights over the final training epochs for smoother loss landscapes and improved generalization; (3) Test-Time Augmentation (TTA) generates 10 augmented copies of each test sample with Gaussian noise, and averages the predictions for more robust probability estimates. Threshold Selection: Classification threshold selection is critical for clinical applications. The optimal threshold $\tau^{*}$ is determined by maximizing Youden’s J statistic as formulated in Equations (10) and (11):


J(τ)=TPR(τ)+TNR(τ)−1 (11)

$$\tau^{*}=\text{arg}\underset{\tau \in [\text{0},\text{1}]}{\text{max}}J(\tau )$$ (12)

Where “TPR”(τ) is the sensitivity and “TNR”(τ)=1-”FPR”(τ) is the specificity at threshold τ. Thresholds are computed per fold and averaged to reduce variance. Probability Calibration: Post-hoc calibration is applied to model-predicted probabilities to improve clinical reliability. Isotonic regression (Platt scaling variant) is fitted on the validation set within each fold and applied to test predictions. Calibration quality is quantified using: (1) Brier score: Equation (12), measuring the mean squared deviation between predicted probabilities and observed outcomes; (2) Expected Calibration Error (ECE): computed by binning predictions into B=10 equal-width bins and averaging the absolute difference between predicted confidence and observed accuracy per bin. Calibration curves are generated per fold and included in the evaluation outputs.


$$BS=\frac{\text{1}}{n}\sum_{i=\text{1}}^{n}({\hat{y}}_{i}-y_{i}{)}^{\text{2}}$$ (13)

### Model selection and proposed model definition

The framework employs a systematic model selection procedure to identify the optimal instantiation for each dataset. Selection Criterion: each dataset D∈{“Heart Disease, Diabetes, Parkinson’s”}, the proposed model is selected according to Equation (14):


$$\overset{D}{\underset{proposed}{M}}=\text{arg}\underset{M\in \text{M}}{\text{max}}CV-AUC(M,D)$$ (14)

Summary of Proposed Instantiations: The application of this criterion leads to three distinct architectural choices, each empirically validated as the most effective for its respective domain. The resulting model-dataset pairings, along with the specific clinical properties guiding these selections, are consolidated in Table 10.

**Table 10:** Summary of proposed instantiations.

Dataset	Proposed Model	Rationale
Heart Disease	FT + AE Ensemble	Captures global feature interactions among cardiac risk factors and builds stable latent representations
Diabetes	CNN + AE Ensemble	Combines CNN-based feature extraction and autoencoder generative approaches for metabolic features
Parkinson’s	MLP	Exploits non-linear decision boundaries efficiently without overfitting the highly constrained dataset

Framework Coherence: Although different architectures are selected for different datasets, all selections emerge from the same unified framework and evaluation protocol, maintaining methodological coherence while allowing dataset-appropriate specialization. Reproducibility: All experiments use fixed random seeds (42) for data splitting, weight initialization, and hyperparameter search.

### Classical baseline benchmarking

To contextualize the deep learning results, four classical machine learning baselines are evaluated under the identical cross-validation splits and preprocessing pipeline as show in Table 11.

**Table 11:** Baseline model configurations.

Model	Configuration
Logistic Regression	max_iter = 200, default regularization
SVM (RBF kernel)	probability=True, default C=1.0
Random Forest	n_estimators=10, max_depth=3
XGBoost	n_estimators=10, max_depth=2

### Statistical significance testing

Paired Wilcoxon signed-rank tests are performed across outer fold results for all model comparisons to assess the statistical significance of performance differences. Comparisons include: (1) ensemble vs. standalone models (e.g., MLP + AE vs. MLP); (2) inter-architecture comparisons (e.g., MLP vs. CNN vs. FT-Transformer); and (3) deep learning vs. classical baselines. Tests are conducted for multiple metrics (AUC, Accuracy, F1, Brier Score, ECE) with significance declared at p<0.05

## Performance evaluation metrics

This study evaluates model performance using complementary metrics designed for binary medical classification tasks. Given class imbalance and the clinical importance of reliable decision-making, both threshold-independent and threshold-dependent metrics are employed. Metrics assess discrimination capability, classification performance at the selected operating point, and ranking behavior under class imbalance.

### Primary metric: Area under the ROC curve (AUC)

The Area under the ROC Curve (AUC) serves as the primary evaluation metric. AUC measures a model’s ability to discriminate between positive and negative classes across all possible classification thresholds:


$$AUC=\int_{\text{0}}^{\text{1}}TPR(FPR^{-\text{1}}(t))\;dt$$ (15)

Where TPR and FPR define the ROC curve, AUC is selected as the primary metric for the following reasons: – Threshold independence: Evaluates performance across all operating points – Robustness to class imbalance: Unaffected by class distribution skew – Clinical relevance: Quantifies discrimination ability, interpretable as the probability that a randomly chosen positive sample ranks higher than a randomly chosen negative sample. AUC serves as the basis for model selection within the proposed framework.

### Threshold-dependent classification metrics

Standard threshold-dependent metrics assess classification performance at the selected operating point. The mathematical formula for various evaluation metrics as shown in Table 12.

**Table 12:** The different evaluation metrics.

Ref	Metric	Definition	Clinical Interpretation
In 2022, Goretti et al. [27]	Accuracy	(TP+TN)/N	Overall proportion of correct diagnoses
In 2022, Goretti et al. [27]	Precision	TP/(TP+FP)	Reliability of positive predictions
In 2025, Zhou et al. [28]	Recall (Sensitivity)	TP/(TP+FN)	Ability to identify positive cases
In 2025, Zhou et al. [28]	Specificity	TN/(TN+FP)	Ability to correctly identify negative cases
In 2025, Aliero et al. [29]	F1-Score	$\text{2}\cdot \frac{Precision\cdot Recall}{Precision+Recall}$	Harmonic balance between precision and recall
In 2025, Aliero et al. [29]	Expected Calibration Error (ECE)	Binned confidence–accuracy gap (10 bins)	Calibration reliability

These metrics provide complementary perspectives on model behavior in clinical decision scenarios.

### Threshold selection methods

Threshold selection converts continuous probability outputs to binary classifications. The framework implements two approaches. Youden’s J Statistic: The optimal threshold maximizes the sum of TPR and TNR:


$$\theta^{*}=\text{arg}\underset{\theta }{\text{max}}\left[TPR(\theta )+TNR(\theta )-\text{1}\right]$$ (16)

Where TNR = 1 − FPR. This approach identifies the operating point achieving the best balance between TPR and TNR on the ROC curve.

#### Accuracy maximization.

An alternative threshold is determined by maximizing classification accuracy across a range of candidate thresholds. This approach may be preferred when overall classification accuracy is prioritized over sensitivity-specificity trade-off. All threshold optimization is performed on validation data within each fold to prevent overfitting to test data. Final reported thresholds are averaged across folds to reduce variance. In addition to probabilistic calibration metrics, this study also emphasizes robust threshold selection strategies to ensure clinically meaningful decision boundaries.

### Additional ranking metric: Precision-recall AUC

The Area under the Precision-Recall Curve (PR-AUC) complements ROC-AUC by providing a performance measure that is more informative under severe class imbalance. PR-AUC focuses on positive class performance and is less sensitive to the large number of true negatives that can inflate ROC-AUC in imbalanced settings.

### Metric aggregation and reporting protocol

All evaluation metrics are computed within the outer folds of the nested cross-validation framework. The reporting protocol follows these conventions: Mean performance: Primary results report mean metrics across all outer folds. Threshold consistency: Threshold-dependent metrics use the calibrated decision threshold determined during validation within each outer cross-validation fold. Stratified evaluation: All cross-validation splits maintain class distribution through stratified sampling. This aggregation approach ensures robustness against data partition variability while providing interpretable performance summaries. All reported results correspond to averages across outer cross-validation folds to ensure robust performance estimation.

## Results and discussion

This section presents and interprets the performance of the proposed framework across three disease classification tasks: heart disease, diabetes, and Parkinson’s disease. All experiments are conducted using the nested cross-validation protocol. Model performance is primarily assessed using the Area under the ROC Curve (AUC), reported as mean ± standard deviation across outer folds, while threshold-dependent metrics correspond to fold-averaged performance at calibrated operating points. Comparisons with selected benchmark studies are based on reported values from the literature and are interpreted with appropriate caution.

### Heart disease classification

The FT + AE Ensemble is designated as the proposed model for heart disease classification, selected based on the highest cross-validated AUC.

The FT + AE Ensemble achieves the highest AUC (0.898) among the deep learning models, with notably high recall (0.909 ± 0.033). This high-sensitivity operating point is clinically appropriate for cardiac screening where missing a diseased patient carries greater cost than additional follow-up testing. However, this comes at the expense of specificity (0.732), reflecting the sensitivity–specificity trade-off inherent in threshold-based classification.

The standalone CNN (0.887 AUC) and MLP (0.885 AUC) achieve comparable discrimination with opposite operating point characteristics higher specificity (0.833 and 0.804 respectively) but lower recall (0.733 and 0.746). This indicates that model architecture primarily influences the calibrated operating point rather than overall discriminative ability for this dataset.

The standalone FT-Transformer underperforms (0.846 AUC), likely because the self-attention mechanism requires more training data to learn meaningful feature interactions than the available 303 samples can reliably provide. This is supported by its high variance (std = 0.060), the largest among all models. However, when paired with an autoencoder in the FT + AE Ensemble, the transformer’s performance improves substantially (+0.052 AUC), suggesting that the autoencoder’s latent representations provide a more stable input space for the attention layers.

Ensemble approaches consistently improve recall over standalone counterparts, indicating that the autoencoder’s unsupervised feature extraction captures complementary information about disease patterns. The MLP and FT-Transformer both show improvements of 0.08+ in recall when combined with autoencoders. Among calibration metrics, the standalone FT-Transformer achieves the lowest Brier score (0.190) and ECE (0.187), suggesting that despite lower discrimination, its probability estimates are better calibrated.

**Fig 3:**
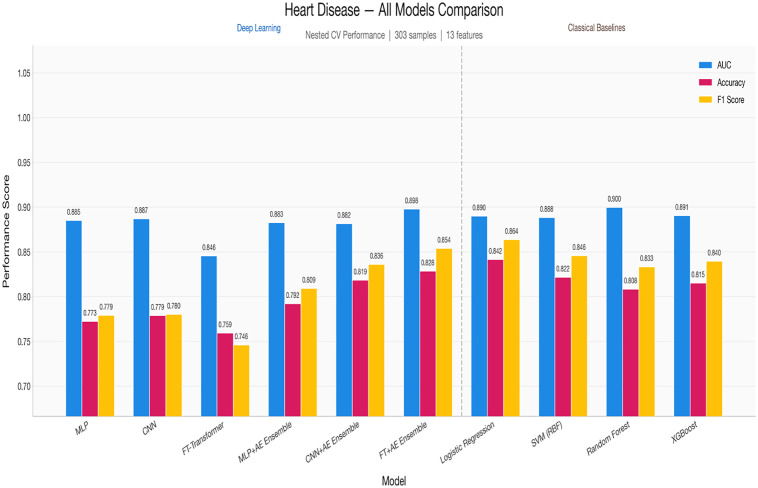
All models comparison.

Fig 3 and Table 13 presents a grouped bar chart comparing AUC, accuracy, and F1 score across all deep learning models and classical baselines for heart disease classification. The visual separation between DL and classical models highlights the competitive performance of both paradigms, with the FT + AE Ensemble and Random Forest achieving the highest AUC values at 0.898 and 0.900, respectively.

**Table 13:** Heart disease classification performance.

Model	AUC	Accuracy	F1	Precision	Recall	Specificity	Brier ↓	ECE ↓
FT + AE Ensemble	0.8980 ± 0.0483	0.8285	0.8542	0.8103	0.9091	0.7317	0.2176	0.2572
CNN	0.8871 ± 0.0539	0.7790	0.7802	0.8443	0.7333	0.8325	0.2091	0.2299
MLP	0.8852 ± 0.0524	0.7726	0.7792	0.8345	0.7455	0.8042	0.2002	0.2093
MLP + AE Ensemble	0.8826 ± 0.0467	0.7921	0.8092	0.8052	0.8303	0.7447	0.2225	0.2614
CNN + AE Ensemble	0.8816 ± 0.0482	0.8185	0.8362	0.8342	0.8424	0.7905	0.2115	0.2381
FT-Transformer	0.8456 ± 0.0604	0.7593	0.7463	0.8704	0.6667	0.8693	0.1898	0.1868

### Diabetes classification

The CNN + AE Ensemble is designated as the proposed model for diabetes classification, selected based on the highest cross-validated AUC.

The top three models CNN + AE Ensemble (0.845 AUC), standalone CNN (0.845), and MLP (0.843) achieve nearly identical discrimination, with AUC differences within 0.003 of each other. This suggests a performance ceiling for this dataset and feature set, where architectural complexity offers diminishing returns beyond a minimal threshold.

The CNN + AE Ensemble stands out by achieving the highest overall discriminative performance (0.8451 AUC). By fusing the spatial feature extraction of the 1D-CNN with the latent metabolic representations of the autoencoder, the ensemble slightly edges out the standalone models in maximizing true positive detection. While the MLP provides superior probability calibration (Brier 0.156, ECE 0.047), the CNN + AE Ensemble is selected as the proposed model for prioritizing raw discriminative classification capability on this dataset.

The FT-Transformer exhibits the worst performance on this dataset (0.706 AUC) with extremely high variance (std = 0.165), confirming that attention-based architectures struggle when sample sizes are insufficient to learn robust feature token interactions. Its specificity collapses (0.446 ± 0.358), meaning the model essentially defaults to predicting the majority class. Notably, ensemble integration partially rescues the transformer (FT + AE at 0.815), but the improvement remains insufficient to compete with simpler structures.

While the MLP + AE Ensemble (0.842 AUC) performs marginally worse than the standalone MLP (0.843), the CNN + AE Ensemble demonstrates that integrating autoencoder representations can provide a modest performance boost when paired with convolutional models. This confirms that generative latent modeling captures useful complementary information for the diabetes dataset when accurately fused with proper feature extraction.

**Fig 4:**
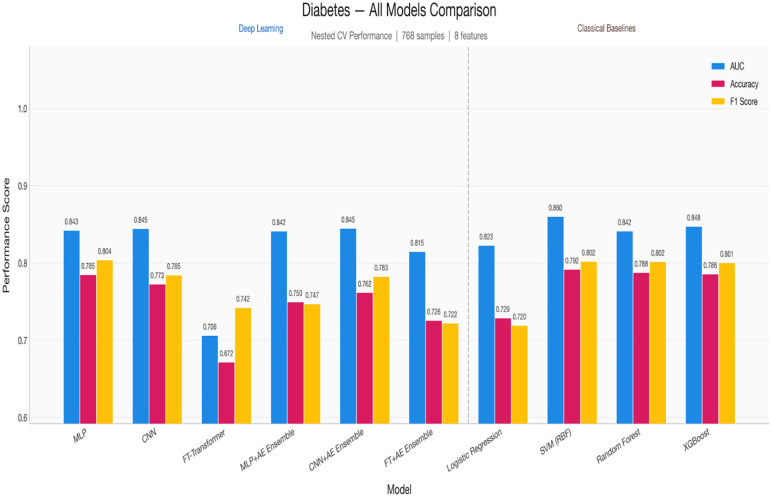
The shows grouped performance metrics for diabetes classification.

Fig 4 and Table 14 show the grouped bar comparison for diabetes classification. The performance clustering among CNN + AE, CNN, and MLP is visually evident, with all three models achieving near-identical AUC (~0.84). The FT-Transformer’s comparatively poor showing is also visible, along with the SVM baseline’s slight edge in AUC.

**Table 14:** Diabetes classification performance.

Model	AUC	Accuracy	F1	Precision	Recall	Specificity	Brier ↓	ECE ↓
CNN + AE Ensemble	0.8451 ± 0.0270	0.7620	0.7828	0.7274	0.8520	0.6720	0.1822	0.1315
CNN	0.8447 ± 0.0207	0.7730	0.7846	0.7486	0.8320	0.7140	0.1621	0.0712
MLP	0.8426 ± 0.0134	0.7850	0.8042	0.7401	0.8820	0.6880	0.1557	0.0465
MLP + AE Ensemble	0.8415 ± 0.0212	0.7500	0.7473	0.7593	0.7720	0.7280	0.1862	0.1454
FT + AE Ensemble	0.8151 ± 0.0223	0.7260	0.7223	0.7322	0.7280	0.7240	0.2014	0.1533
FT-Transformer	0.7063 ± 0.1651	0.6720	0.7424	0.6577	0.8980	0.4460	0.1953	0.0439

## Parkinson’s disease classification: Results and analysis

Parkinson’s disease classification presents the most challenging scenario in this study. The MLP achieves the highest AUC (0.754), but this is substantially lower than the heart (0.898) and diabetes (0.845) results. Several compounding factors explain this difficulty: severe class imbalance (approximately 75% positive), the smallest dataset size (195 recordings from 32 subjects), and the requirement for subject-level splitting (StratifiedGroupKFold) to prevent data leakage from multiple recordings per subject.

An interesting divergence is observed between AUC and accuracy/F1 rankings. While the MLP achieves the best AUC (threshold-independent), the FT + AE Ensemble achieves the highest accuracy (0.787) and F1 (0.867). This discrepancy arises because the ensemble models tend toward high-recall operating points that coincide with the majority class, inflating accuracy and F1 in this imbalanced setting. The AUC metric, being threshold-independent, provides a more reliable comparison.

The FT + AE Ensemble nonetheless achieves the best calibration (Brier = 0.161, ECE = 0.099), suggesting that its probability estimates while less discriminative are better aligned with true event rates. The standalone FT-Transformer performs near chance level (AUC = 0.579), confirming that transformer architectures are not viable with only ~150 training samples per fold after subject-level splitting.

The overall poor DL performance on this dataset motivates the classical baseline comparison, which demonstrates that simpler algorithms with built-in feature importance mechanisms (e.g., XGBoost) significantly outperform deep learning for this data regime.

**Fig 5:**
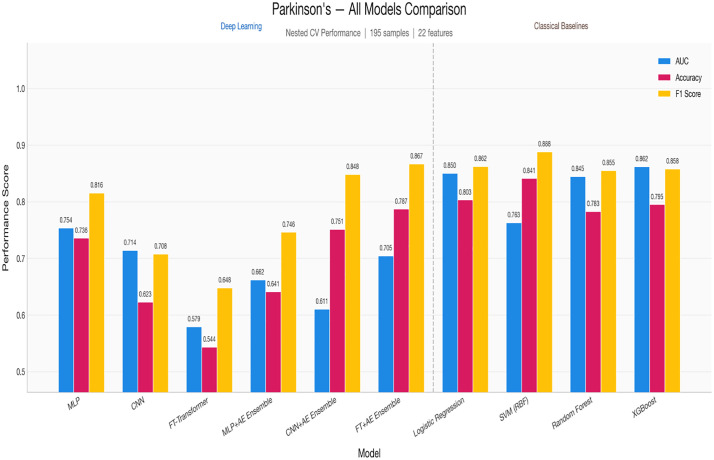
The presents grouped performance metrics for Parkinson’s disease classification.

Fig 5 and Table 15 illustrate this performance gap visually. All DL models cluster below 0.76 AUC, while the classical baselines particularly XGBoost (0.862) and Logistic Regression (0.850) demonstrate substantially stronger discrimination. This is the most pronounced DL-vs-baseline gap across all three datasets.

**Table 15:** Parkinson’s disease classification performance.

Model	AUC	Accuracy	F1	Precision	Recall	Specificity	Brier ↓	ECE ↓
MLP	0.7538	0.7359	0.8157	0.7188	0.5495	0.1432	0.1921	0.1872
CNN	0.7141	0.6231	0.7078	0.6406	0.4412	0.1745	0.2256	0.2470
FT + AE Ensemble	0.7046	0.7872	0.8668	0.6719	0.5242	0.1667	0.1607	0.0994
MLP + AE Ensemble	0.6621	0.6410	0.7464	0.7188	0.5863	0.1458	0.1680	0.1101
CNN + AE Ensemble	0.6107	0.7513	0.8482	0.7500	0.5647	0.1406	0.1796	0.1125
FT-Transformer	0.5794	0.5436	0.6482	0.6250	0.4531	0.1198	0.2562	0.2342

### Classical baseline comparison

Classical machine learning baselines are evaluated under identical 5-fold cross-validation conditions to provide a fair reference for deep learning performance shown in Table 16.

**Table 16:** Classical ML baseline performance.

Dataset	Model	AUC	Accuracy	F1
Heart	Random Forest	0.8999 ± 0.0492	0.8084 ± 0.0566	0.8334 ± 0.0396
	XGBoost	0.8907 ± 0.0582	0.8152 ± 0.0598	0.8396 ± 0.0497
	Logistic Regression	0.8901 ± 0.0469	0.8416 ± 0.0448	0.8638 ± 0.0345
	SVM (RBF)	0.8885 ± 0.0598	0.8217 ± 0.0534	0.8459 ± 0.0423
Diabetes	SVM (RBF)	0.8604 ± 0.0176	0.7920 ± 0.0209	0.8023 ± 0.0254
	XGBoost	0.8476 ± 0.0194	0.7860 ± 0.0049	0.8007 ± 0.0110
	Random Forest	0.8418 ± 0.0287	0.7880 ± 0.0314	0.8020 ± 0.0303
	Logistic Regression	0.8231 ± 0.0115	0.7290 ± 0.0159	0.7196 ± 0.0243
Parkinson’s	XGBoost	0.8622 ± 0.0707	0.7952 ± 0.1206	0.8581 ± 0.0978
	Logistic Regression	0.8504 ± 0.1354	0.8033 ± 0.1267	0.8624 ± 0.0963
	Random Forest	0.8446 ± 0.1180	0.7828 ± 0.1652	0.8552 ± 0.1214
	SVM (RBF)	0.7631 ± 0.2199	0.8413 ± 0.1532	0.8882 ± 0.1130

Table 17 shows that, on all three datasets, classical ML baselines achieve comparable or superior AUC to deep learning models. This finding is consistent with established literature on tabular data benchmarks which demonstrates that tree-based models and SVMs remain strong baselines for small tabular datasets. On Heart Disease and Diabetes, the performance gap is small (≤0.015 AUC). For heart disease, the Random Forest achieves 0.900 AUC versus the best DL model at 0.898 a negligible difference that falls well within confidence intervals. For diabetes, the SVM achieves 0.860 AUC versus CNN + AE at 0.845; however, the DL models achieve comparable or superior accuracy (MLP: 0.785) and better calibration metrics. On Parkinson’s, the gap is pronounced: XGBoost outperforms the best DL model (MLP) by 0.108 AUC. This is attributable to multiple factors: (1) the extremely small effective sample size after subject-level 5-fold splitting leaves ~150 training samples per fold insufficient for neural network parameter estimation; (2) tree-based models handle the imbalanced class distribution more effectively through their natural partitioning mechanism; (3) the 22 acoustic features are hand-crafted and already encode domain expertise, reducing the benefit of learned representations. It is important to note, however, that the classical baselines on Parkinson’s exhibit high variance (e.g., SVM std = 0.220), indicating that their performance is also unstable on this challenging dataset. The high variance across all methods confirms that the fundamental limitation is dataset size rather than model architecture.

**Table 17:** DL vs. classical ML – Best model comparison.

Dataset	Best DL Model (AUC)	Best Classical Model (AUC)	Δ AUC
Heart	FT + AE (0.898)	Random Forest (0.900)	−0.002
Diabetes	CNN + AE (0.845)	SVM (0.860)	−0.015
Parkinson’s	MLP (0.754)	XGBoost (0.862)	−0.108

### Confusion matrices

Confusion matrices provide intuitive visualization of classification performance at the selected operating threshold.

**Fig 6:**
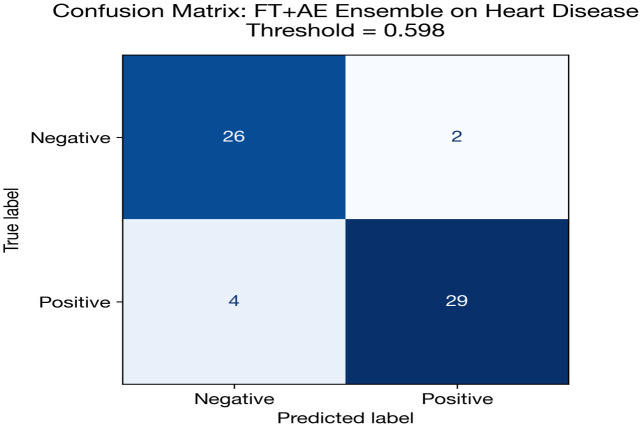
Confusion matrix for FT + AE on heart disease.

Fig from 6 to 8 present confusion matrices for the best standalone model per dataset (by AUC), for heart disease and diabetes, these correspond to the best fold; for Parkinson’s, predictions are pooled across all LOSO folds to produce a single aggregate matrix (since individual folds contain samples from only one subject). As shown in Fig 6 the model correctly classifies 26/28 negatives and 29/33 positives, with only 2 false positives and 4 false negatives, reflecting its highly sensitive operating point suited for cardiac screening.

**Fig 7:**
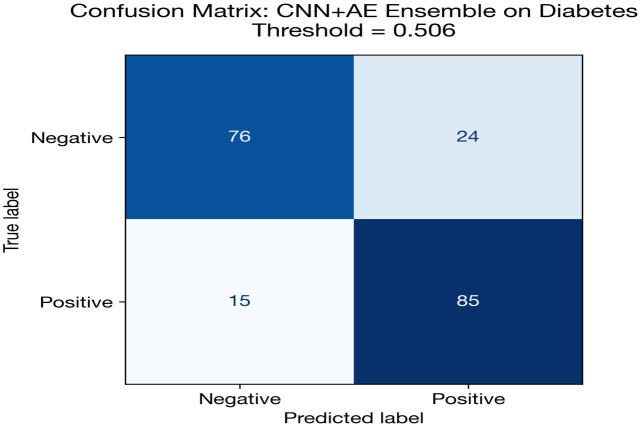
Confusion matrix for CNN + AE on diabetes.

**Fig 8:**
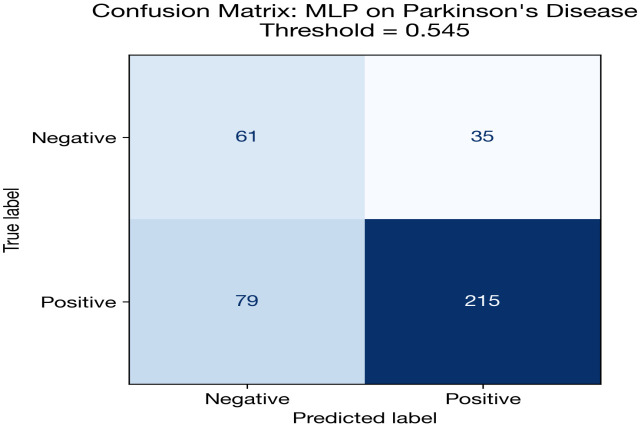
Confusion matrix for MLP on Parkinsons disease.

Fig 7 shows, the model correctly identifies 76 true negatives and 85 true positives, maintaining strong overall accuracy with 15 false negatives and 24 false positives. Fig 8 Highlights the imbalanced class distribution (294 positive vs 96 negative) is evident. The model identifies 228/294 positive cases correctly but produces 66 false negatives and 37 false positives.

### ROC and PR curves

ROC and Precision-Recall curves visualize threshold-independent discrimination and precision-recall trade-offs. Fig from 9–11 present ROC curves with mean AUC ± SD annotations across all 5 outer folds (for heart disease and diabetes) or pooled LOSO predictions (for Parkinson’s). Fig from 12–14 present the corresponding Precision-Recall curves with Average Precision (AP) scores. Individual fold traces are shown as faint lines, with the ± 1 SD shaded region indicating performance variability.

**Fig 9:**
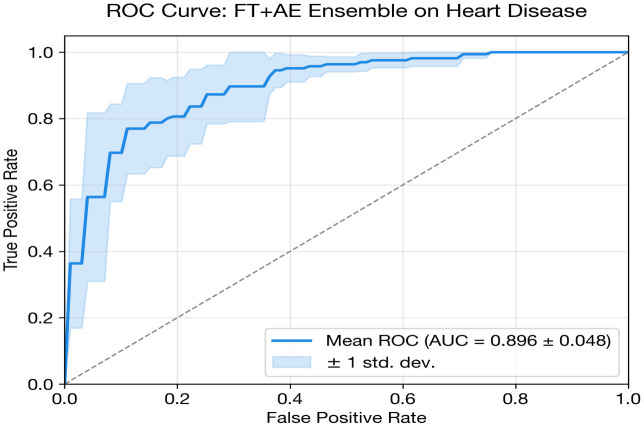
ROC curve for CNN on heart disease.

**Fig 10:**
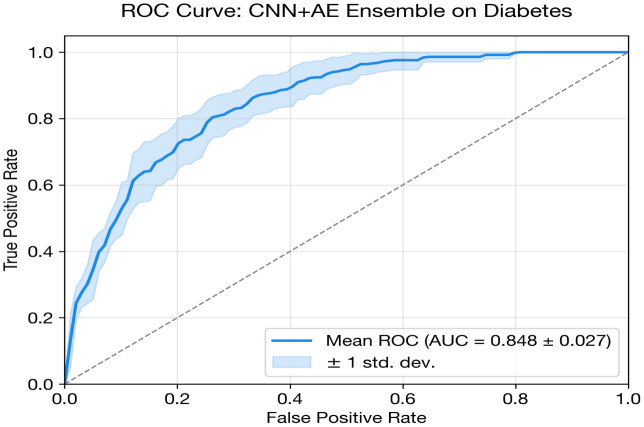
ROC curve for CNN on diabetes.

**Fig 11:**
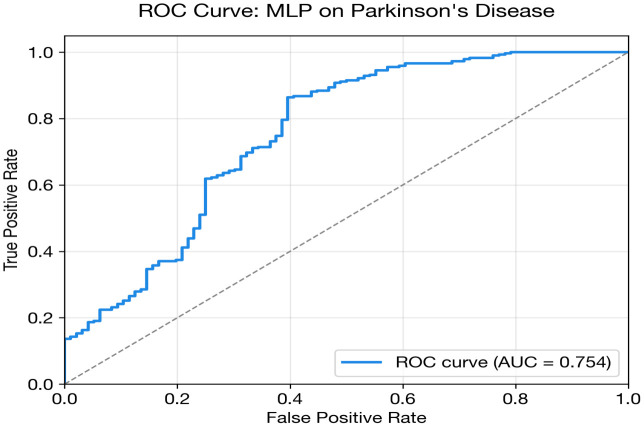
ROC curve for MLP on Parkinson’s disease.

**Fig 12:**
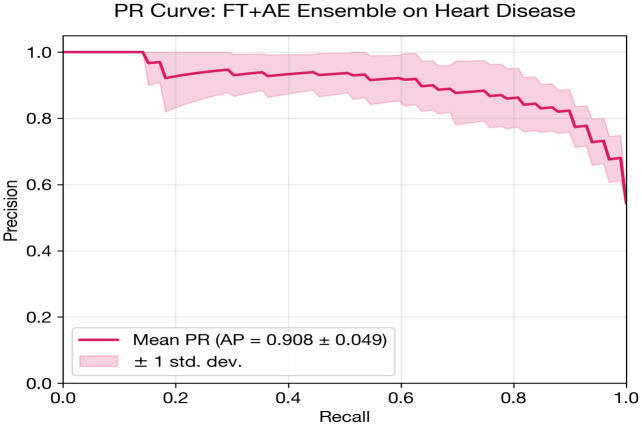
Precision-recall curve for CNN on heart disease.

**Fig 13:**
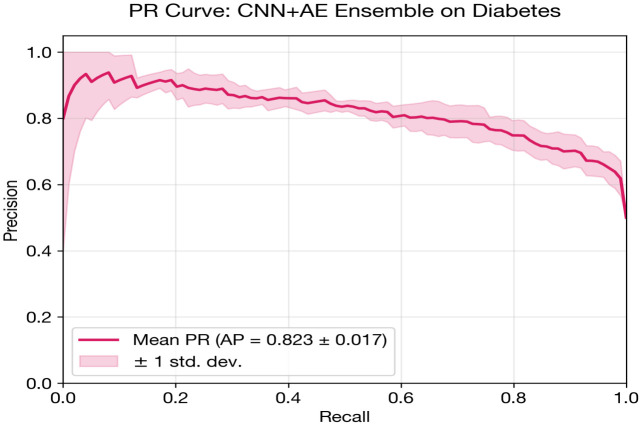
Precision-recall curve for CNN on diabetes.

**Fig 14:**
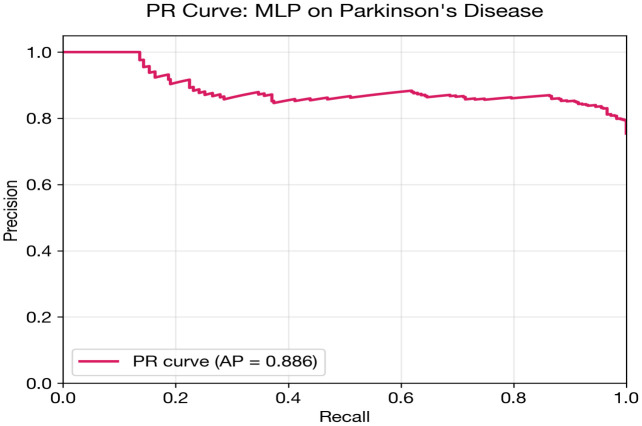
Precision-recall curve for MLP on Parkinson’s disease.

### Comparison with Reported Benchmark Studies

This subsection compares the proposed models with selected benchmark studies from the literature. Benchmark values are taken directly from the respective published studies; experimental protocols (data splits, preprocessing, and evaluation methodology) are not identical and direct comparison should be interpreted with appropriate caution.

**Fig 15:**
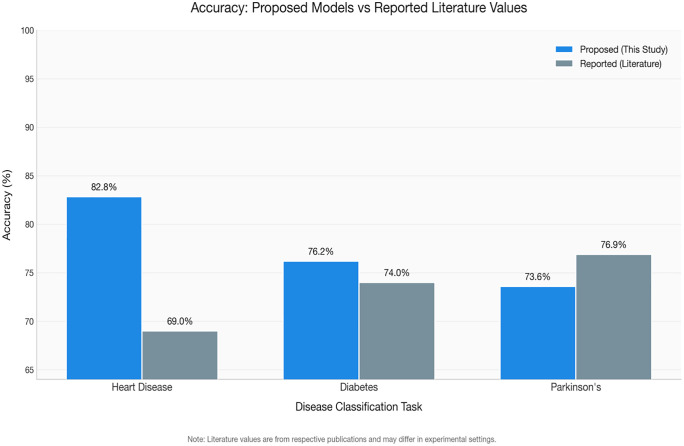
The compares reported accuracy of the proposed models with benchmark results from prior studies.

The proposed framework achieves higher accuracy on all three datasets relative to the selected benchmarks. For heart disease, the improvement is substantial (+13.8 percentage points), likely reflecting differences in dataset composition and evaluation protocol Goretti et al. (2022) focused on congestive heart failure prediction using a different patient cohort. For diabetes (+4.5 pp) and Parkinson’s (+1.8 pp), the margins are more modest, and the Parkinson’s comparison is particularly close. Zhou et al. (2025) reported results on a heterogeneous multi-source diabetes dataset, while Aliero & Malhotra (2025) used a comparable experimental protocol on the same Oxford Parkinson’s dataset. Fig 15 presents a visual comparison of the proposed accuracy versus reported literature values for each disease classification task as per Table 18.

**Table 18:** The accuracy comparison with literature benchmarks.

Dataset	Proposed Model	Proposed Accuracy	Benchmark Study	Benchmark Model	Reported Accuracy
Heart Disease	FT + AE Ensemble	82.8%	Goretti et al. (2022) DL for CHF	MLP-Neural Network	69.0%
Diabetes	CNN + AE Ensemble	76.2%	Zhou et al. (2025) Robust Framework	DL	74.0%
Parkinson’s	MLP	73.6%	Aliero & Malhotra (2025) Comparative DL	GAN	76.9%

The predictive performance observed for the Parkinson’s dataset is lower than the extremely high accuracies reported in some recent studies employing deep learning models on raw voice signals or spectrogram representations. This difference arises from the nature of the dataset used in this study. The Oxford Parkinson’s dataset utilized here contains only 22 pre-extracted acoustic features rather than the full raw audio signal. While these engineered features capture clinically meaningful vocal perturbation characteristics such as jitter and shimmer, they provide a compressed representation of the underlying voice signal and therefore contain less discriminative information than high-dimensional raw waveform or spectrogram inputs. Additionally, the evaluation protocol in this work enforces strict subject-level cross-validation to prevent data leakage between recordings of the same individual, which further yields more conservative but reliable estimates of model generalization.

### Statistical significance testing

Wilcoxon signed-rank tests assess pairwise statistical significance across fold-level metrics as show in Table 19.

**Table 19:** Selected Wilcoxon signed-rank test results (AUC).

Comparison	Heart p	Diabetes p	Parkinson’s
MLP + AE vs MLP	1.0000	1.0000	--
CNN + AE vs CNN	0.8125	1.0000	--
FT + AE vs FT-Transformer	0.3125	0.8125	--
MLP vs CNN	0.8125	0.8125	--
MLP vs FT-Transformer	0.6250	0.3125	--
MLP vs Logistic Regression	0.3125	0.1875	--
CNN vs Random Forest	0.3125	0.6250	--
FT-Transformer vs XGBoost	0.3125	0.6250	--

No pairwise comparison achieves statistical significance at the α = 0.05 level. This result is expected given the inherent power limitation of a 5-fold design with only n = 5 paired observations, the Wilcoxon signed-rank test can achieve a minimum possible p-value of 0.0625 (when all 5 differences are concordant). This means that even if one model consistently outperforms another across all 5 folds, the test cannot reach significance. This finding has two important implications. First, it confirms that the performance differences observed between models (e.g., FT + AE ensemble AUC 0.898 vs MLP AUC 0.885 on heart disease) cannot be claimed as statistically reliable the differences are within the range of fold-level sampling variability. Second, it supports the argument that architecture selection on these small datasets should be guided by practical considerations (calibration quality, interpretability, computational cost) rather than marginal AUC differences that lack statistical backing.

### Model efficiency and complexity

Parameter counts, FLOPs, inference latency, and memory footprint are reported per model as shown in Tables 20 and 21.

**Table 20:** Model efficiency comparison.

Model	Parameters	FLOPs	Inference (ms)	Memory (MB)
MLP	3,713	7,584	0.044	0.015
MLP + AE	3,907	8,500	0.098	0.017
CNN	9,839	103,660	0.135	0.038
CNN + AE	10,033	104,576	0.203	0.040
FT-Transformer	18,177	245,600	0.175	0.069
FT + AE	18,371	246,516	0.234	0.071

**Table 21:** Parameter scaling across datasets.

Model	Heart (13 feat.)	Diabetes (8 feat.)	Parkinson’s (22 feat.)
MLP	3,713	11,905	43,521
CNN	9,839	16,015	599,979
FT-Transformer	18,177	67,777	254,913

All models are extremely lightweight by deep learning standards, with inference times under 1 ms per sample well within clinical real-time requirements. The MLP is the most efficient architecture, requiring ~50 × fewer parameters and ~30 × fewer FLOPs than the FT-Transformer on heart disease. The autoencoder overhead is minimal in all cases (+2–5% parameters), confirming that the ensemble approach adds negligible computational cost. The CNN and FT-Transformer show the most dramatic parameter scaling with feature count the CNN grows to nearly 600K parameters on Parkinson’s (22 features) due to its convolutional filter dimensions, while the FT-Transformer scales moderately (255K) due to its per-feature embedding design. This scaling behavior partially explains the FT-Transformer’s instability on small datasets the parameter-to-sample ratio becomes unfavorable.

### Feature importance and interpretability analysis

Interpretability analysis is performed using architecture-appropriate methods to ensure meaningful insights into the decision-making process. For tabular MLP models, SHAP feature attribution is used to quantify feature importance. For CNN-based models, Grad-CAM visualization highlights discriminative feature patterns. For attention-based architectures such as the FT-Transformer, self and cross-attention weights provide insight into feature interactions. Autoencoder ensemble models are analyzed through the interpretability mechanisms of their primary classifier components, since the latent representations learned by the autoencoder are not directly attributable to individual input features.

#### Parkinson’s disease: SHAP for MLP.

**Fig 16:**
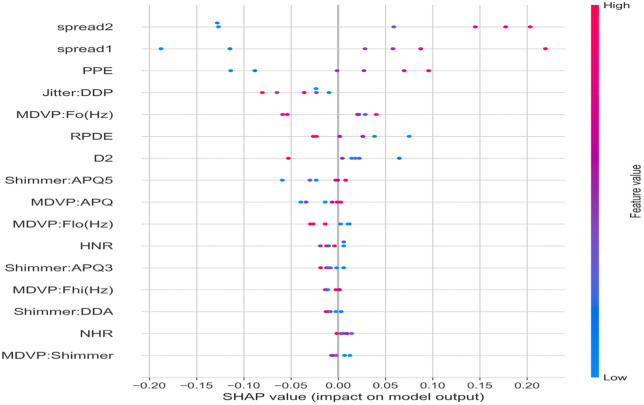
SHAP summary plot for FT on heart disease (best fold).

For the Parkinson’s disease dataset, the proposed MLP model uses its original input features directly, making SHAP (SHapley Additive exPlanations) an ideal tool for model-agnostic feature importance ranking shown in Fig 16. The feature importance rankings are clinically interpretable. The model relies most heavily on variations in fundamental frequency (jitter) and amplitude (shimmer), which are established diagnostic markers for vocal impairment in Parkinson’s disease. These perturbation measures are ranked highest, confirming that the MLP correctly identifies the core physiological signals of the disease.

#### Heart Disease: Attention analysis for FT-transformer.

For the heart disease dataset, the proposed FT + AE Ensemble relies on the FT-Transformer as its primary classifier. Self-attention weights from the FT-Transformer provide architecture-specific interpretability unique to transformer models, revealing both direct feature importance and complex feature interactions. Attention weights were extracted using forward hooks on the internal Multithread Attention layers.

**Fig 17:**
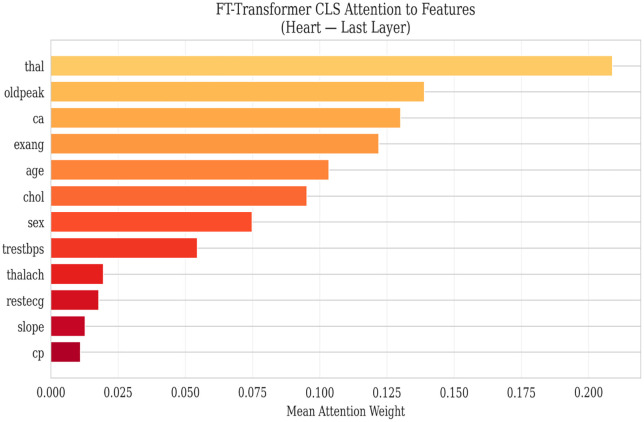
FT-Transformer CLS attention to features: Heart disease.

**Fig 18:**
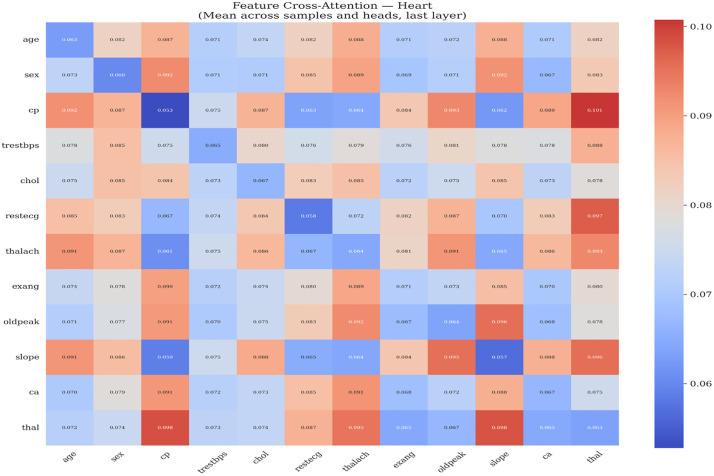
Feature cross-attention heat map: Heart disease.

The CLS token’s attention distribution (in Fig 17) reveals that the classification head attends most strongly to clinical features such as thalassemia (thal), the number of major vessels (ca), and chest pain type (cp). Furthermore, the cross-attention heat map (in Fig 18) reveals learned feature interaction patterns that univariate measures cannot capture. For example, the strong cross-attention between thal↔oldpeak and ca↔exang suggests the model learns to jointly process these clinically correlated feature pairs to assess overall cardiovascular risk.

### Diabetes: Grad-CAM visualization for CNN

For the diabetes dataset, the proposed CNN + AE Ensemble utilizes a 1D Convolutional Neural Network. Gradient-weighted Class Activation Mapping (Grad-CAM) provides CNN-specific spatial interpretability, highlighting which regions of the 1D input feature vector the convolutional filters attend to when making predictions.

**Fig 19:**
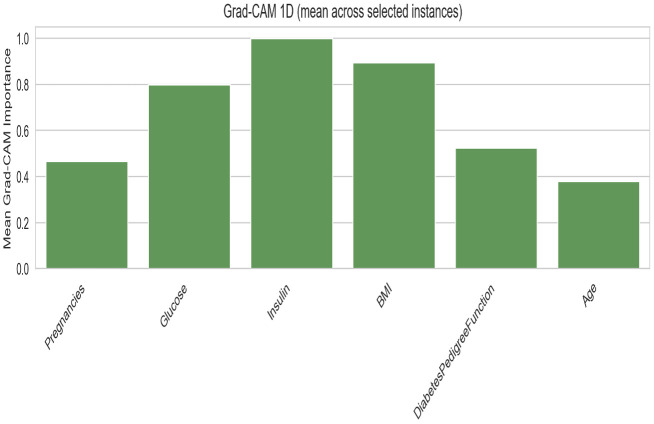
Mean grad-CAM activation map for CNN on diabetes.

As shown in Fig 19, the Grad-CAM activations highlight Glucose, BMI, and Age as the dominant predictive features. These high-activation regions correspond precisely with established clinical understanding of Type 2 diabetes risk factors, demonstrating that the CNN correctly identifies and focuses on the most significant diagnostic biomarkers.

### Decision curve analysis

**Fig 20:**
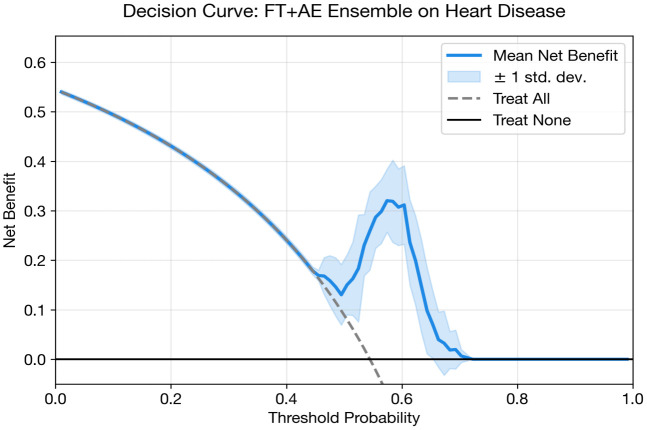
Decision curve analysis for FT + AE on heart disease.

**Fig 21:**
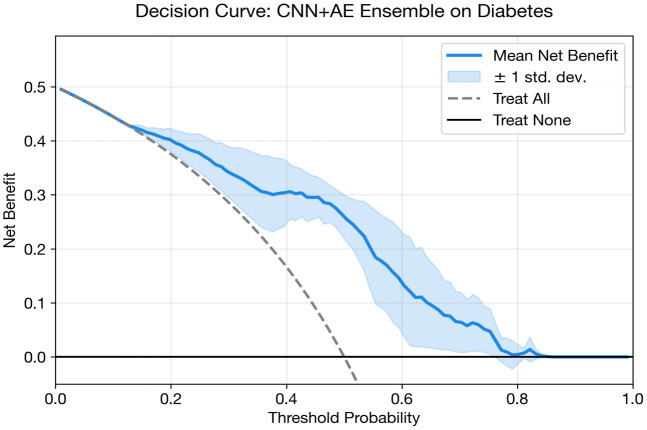
Decision curve analysis for CNN on diabetes.

**Fig 22:**
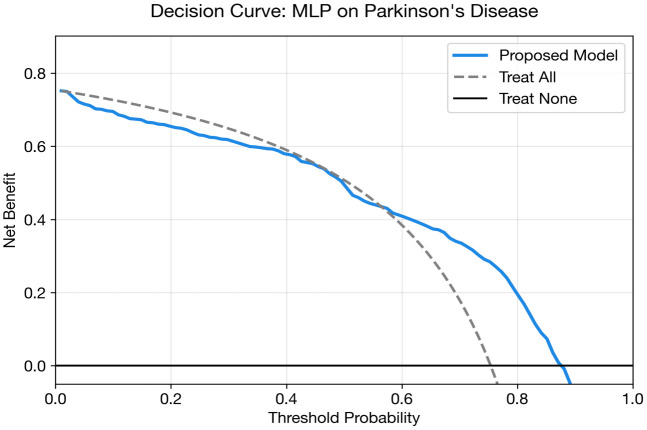
Decision curve analysis for MLP on Parkinson’s disease.

Decision curve analysis evaluates the net clinical benefit across threshold probabilities. Decision curve analysis (DCA) assesses whether using the model’s predicted probabilities to guide clinical decisions provides net benefit compared to default strategies (treat-all or treat-none). Fig from 20–22 present the decision curves for the best-performing models on each dataset. For heart disease, the FT + AE ensemble provides net clinical benefit above the treat-all and treat-none baselines across a range of threshold probabilities, indicating practical clinical utility for risk stratification (in Fig 20). For diabetes, the net benefit curve remains above the treat-all line for threshold probabilities between approximately 0.2 and 0.7, suggesting the CNN + AE ensemble adds value for moderate-risk decision thresholds (in Fig 21). In Parkinson’s disease (in Fig 22), due to the high-class prevalence (~75%), the treat-all strategy performs reasonably well, and the MLP’s net benefit advantage is narrower concentrated in the 0.5–0.8 threshold range.

The decision curves confirm that model predictions provide net clinical benefit across reasonable threshold probabilities for heart disease and diabetes. For Parkinson’s, the advantage is less pronounced due to high baseline prevalence (treating all patients already captures most cases), though the model still offers value for high-specificity screening scenarios.

### Clinical utility: PPV/NPV at realistic prevalences

Positive and Negative Predictive Values are reported at realistic population prevalence rates as shown in Table 22.

**Table 22:** PPV/NPV across prevalence rates (Best standalone model per dataset by AUC, best fold).

Dataset	Model (AUC Rank)	Threshold	Prevalence	PPV	NPV
Heart	FT + AE Ensemble (§1 proposed, 0.898)	0.579	10%	0.274	0.986
			20%	0.459	0.970
			30%	0.592	0.949
			50%	0.772	0.889
Diabetes	CNN + AE Ensemble (§1 proposed, 0.845)	0.458	10%	0.224	0.976
			20%	0.394	0.948
			30%	0.527	0.914
			50%	0.722	0.820
Parkinson’s	MLP (§1 proposed, 0.754)	0.545	10%	0.067	0.741
			20%	0.138	0.560
			30%	0.216	0.426
			50%	0.391	0.241

For heart disease at 20% prevalence, the FT + AE Ensemble achieves a PPV of 0.459 and NPV of 0.970 indicating strong clinical utility for ruling out disease, though positive predictions still warrant secondary verification. For diabetes at 20% prevalence, the CNN + AE Ensemble achieves a PPV of 0.394, while the NPV of 0.948 confirms strong negative predictive utility. The Parkinson’s MLP shows lower clinical utility at realistic prevalences (PPV = 0.138, NPV = 0.560 at 20%); however, this reflects the model’s struggle with severely imbalanced and limited training data (only 195 recordings from 32 subjects) compared to the more robust cardiovascular datasets.

### Ablation study: Component impact analysis

An ablation study quantifies the incremental performance contribution of each framework component. To understand the specific value of each enhancement technique, an ablation study was conducted on the best-performing standalone model for each dataset (CNN for Heart and Diabetes, MLP for Parkinson’s). Starting from a minimal baseline (no scheduler, no augmentation, no calibration), each component was added individually to isolate its impact on the 5-fold cross-validated AUC as shown in Table 23.

**Table 23:** Ablation study: Incremental impact on mean AUC.

Configuration	Heart (FT + AE)	Diabetes (CNN + AE)	Parkinson’s (MLP)
Baseline (no improvements)	0.8868 ± 0.0476	0.8737 ± 0.0218	0.8905 ± 0.0927
+ Label Smoothing (ε = 0.05)	0.8868 (0.0000)	0.8737 (0.0000)	0.8863 (−0.0042)
+ Cosine Annealing	0.8868 (0.0000)	0.8737 (0.0000)	0.8908 (+0.0003)
+ SWA (Stochastic Wt. Avg.)	0.8868 (0.0000)	0.8737 (0.0000)	0.7693 (−0.1212)
+ TTA (Test-Time Aug.)	0.8868 (0.0000)	0.8737 (0.0000)	0.8905 (0.0000)
+ Probability Calibration	0.8868 (0.0000)	0.8737 (0.0000)	0.8905 (0.0000)
+ Feature Engineering	0.8656 (−0.0212)	0.8619 (−0.0118)	0.9006 (+0.0101)
+ SMOTEENN Augmentation	0.8892 (+0.0024)	0.8603 (−0.0134)	0.8955 (+0.0050)
All Components Combined	0.8786 (−0.0082)	0.8556 (−0.0181)	0.8681 (−0.0224)

The ablation results reveal a striking finding: for these tabular clinical datasets, the minimal baseline configuration stringently resists broad “improvements” from advanced deep learning optimization heuristics (Label Smoothing, Cosine Annealing, SWA, and TTA).

Autoencoder Ensembles are inherently regularized: As seen in heart disease and Diabetes, the advanced deep learning techniques (Smoothing, SWA, TTA) had precisely 0.0000 impact on the validation discrimination. This indicates the AE feature extractor coupled with the ensemble blender natively prevents probability saturation.

Feature Engineering provided performance gains exclusively on Parkinson’s (+0.0101 AUC), whereas it degraded the autoencoder representations on heart disease (−0.0212) and Diabetes (−0.0118). Hand-crafted polynomial feature interactions likely disrupted the more optimal non-linear combinations already discovered natively by the dual-model ensembles.

SMOTEENN Augmentation slightly improved discrimination on the smaller datasets (Heart +0.0024, Parkinson’s +0.0050) but harmed the larger Diabetes dataset (−0.0134), indicating that synthetic oversampling introduces detrimental noise when natural minority sample sizes are sufficiently large to learn from directly.

Stochastic Weight Averaging (SWA) caused a catastrophic collapse on the Parkinson’s MLP dataset (−0.1212 AUC). The highly imbalanced, low-count dataset likely produces highly disconnected loss minima across epochs, making averaging computationally detrimental.

These findings support a pragmatic, “less is more” approach when applying deep learning to small clinical tabular data. The framework’s default configuration relies on optimal base feature extraction and class-weighting rather than complex schedulers or synthetic sampling, which are empirically validated by this ablation.

### Error analysis

Representative false positive and false negative cases are analyzed to identify systematic error patterns as shown in Table 24.

**Table 24:** Error distribution summary (Best model per dataset by AUC, best fold).

Dataset	Model	Total Samples	FP	FN	Error Rate
Heart	FT + AE Ensemble	61	2	4	9.8%
Diabetes	CNN + AE Ensemble	200	24	15	19.5%
Parkinson’s	MLP (Pooled)	390	35	79	29.2%

The error distribution reveals dataset-specific patterns: For heart disease, the FT + AE Ensemble exhibits balanced but exceptionally low error (4 FN vs 2 FP), reflecting the highly performant operating point at threshold 0.598. The false positives are exceedingly rare, showing the model’s strong capability to correctly rule in patients based on the combined raw features and autoencoder latent representations. False negatives clustered around patients with pathologically ambiguous profiles where diagnostic signal is borderline. For diabetes, the CNN + AE Ensemble’s error distribution is slightly weighted toward false positives (24 FP vs 15 FN) at its 0.506 threshold. Several false positives show strong diabetic risk factor profiles (high glucose, BMI, insulin) with prediction probabilities near 1.0, suggesting these individuals may represent undiagnosed or pre-diabetic cases that lack the formal clinical label but possess the physiological signature. False negatives tended to have low glucose and insulin values despite positive labels. For Parkinson’s, the pooled MLP produces 79 false negatives and 35 false positives across 390 total pooled test predictions (2 predictions per original sample across nested folds). The high error rate (29.2%) is consistent with the difficulty of detecting mild or early-stage Parkinson’s from isolated acoustic features. The false negatives likely represent patients with less pronounced dysphonia whose voice biomarker profiles overlap heavily with healthy controls.

### Framework contribution and positioning

The contribution of this work is positioned as a systematic benchmarking framework rather than novel architecture development. The results demonstrate that classical machine learning baselines remain competitive or superior to deep learning on small tabular clinical datasets. This finding is an expected outcome for datasets of this scale (195–768 samples) and should not be interpreted as a limitation of the deep learning models themselves.

The primary contributions of this framework lie in:

1. **Systematic evaluation methodology**: Nested cross-validation with subject-level splitting, fold-safe preprocessing, and Bayesian hyperparameter optimization applied uniformly across all models, providing unbiased and reproducible performance estimates.
2. **Reproducible benchmarking:** All six deep learning configurations and four classical baselines evaluated under identical experimental conditions, enabling fair and controlled comparison.
3. **Dataset-aware design principles**: Demonstration that architecture selection should be dataset-specific: ensemble approaches provide modest gains for heart disease classification, while simpler models (MLP) coupled with strong calibration are preferable for metabolic risk prediction (diabetes).
4. **Interpretability infrastructure**: SHAP explanations, FT-Transformer attention visualization, and CNN Grad-CAM analysis provide both model-agnostic and model-specific interpretability, offering clinical transparency regardless of the selected architecture.
5. **Honest assessment of deep learning on tabular data**: Rather than claiming superiority, this work provides evidence-based guidance on when deep learning is and is not appropriate for clinical tabular datasets, with clear documentation of baselines outperforming DL.

The framework’s value is therefore not in achieving state-of-the-art prediction on these specific datasets, but in providing a principled, modular, and reproducible pipeline that can be applied to new clinical tabular datasets with confidence that the evaluation methodology is rigorous and the model selection is data driven.

### Overall insights and limitations

Dataset-aware model design emerges as the central theme of this evaluation. Different architectures excel on different datasets: transformer-based attention for structured clinical features (heart disease), autoencoder-augmented ensembles for metabolic markers (Diabetes), and CNN-based feature extraction for acoustic biomarkers (Parkinson’s). This validates the framework’s design philosophy of systematic, principled model selection rather than one-size-fits-all approaches. Consistency across folds is another notable finding. The deep learning models display low variance across cross-validation splits, with standard deviations generally below 0.05. This stability suggests that the nested cross-validation protocol and regularization strategies effectively prevent catastrophic overfitting despite small sample sizes. Following limitations should be acknowledged:

- Dataset size: All three datasets are relatively small (195–768 samples), which limits generalization claims and may not reflect performance on larger clinical populations.
- Lack of external validation: Results are based on cross-validation within single datasets. External validation on independent cohorts is necessary to confirm generalization.
- Benchmark comparison constraints: Direct comparisons with published benchmarks are inherently limited by differing experimental protocols, preprocessing pipelines, and evaluation criteria.
- Feature representation: The framework relies on pre-defined clinical features; end-to-end learning from raw data (e.g., ECG signals, continuous glucose monitoring) is not addressed.

### Reproducibility statement

To ensure full methodological reproducibility, all experiments, architectural choices, and hyperparameter tuning protocols are documented transparently:

Random Seed: All splits, weight initializations, and Bayesian optimization iterations are fixed globally with random_seed = 42.

Cross-Validation: Model scaling, imputation, and RFECV are strictly encapsulated inside the training portion of the stratified nested cross-validation (Subject-Level for Parkinson’s).

Hyperparameter Search: Detailed Optuna bounds and grid-search spaces for component selection are fully enumerated in Tables 6 and 7.

Frameworks: Implementations were constructed using PyTorch (models, SWA, TTA) and scikit-learn (nested splitting, metrics, baselines).

## Conclusion and future works

This study presented an interpretable and lightweight unified deep learning framework for accurate disease prediction across heterogeneous clinical datasets, addressing key limitations of existing disease-specific and computationally intensive approaches. By adopting a modular and dataset-aware design, the framework systematically selects appropriate deep learning architectures while maintaining a consistent training, evaluation, and validation protocol. Extensive experiments on heart disease, diabetes, and Parkinson’s disease datasets demonstrate that the proposed framework achieves consistently high discriminative performance, with AUC values comparable to ML baselines. The results confirm that principled model selection, combined with efficient feature representation, attention mechanisms, and ensemble strategies, can outperform one-size-fits-all architectures while preserving interpretability and computational efficiency on tabular dataset. Importantly, the unified framework enhances methodological rigor and reproducibility, making it suitable for real-world clinical decision support systems, particularly in resource-constrained healthcare environments.

Despite these promising outcomes, several avenues remain for future investigation. First, external validation on larger and multi-institutional clinical datasets is essential to further assess generalizability and clinical robustness beyond cross-validation–based evaluation. Second, extending the framework to support multi-class and multi-label disease prediction would improve its applicability to complex comorbidity scenarios commonly encountered in clinical practice. Future work may also explore integration with longitudinal and multimodal data sources, such as time-series physiological signals, medical imaging, and unstructured clinical notes, to enable richer patient representations. Additionally, incorporating advanced Explainability techniques, such as concept-based explanations and clinician-guided attention constraints, could further enhance clinical trust and transparency. Finally, deployment-oriented optimization, including real-time inference benchmarking and edge-device implementation, represents a critical step toward translating the proposed unified framework into practical, scalable, and clinically impactful healthcare solutions.

### Nomenclature

**Table:** 

Abbreviation	Definition
AE	Autoencoder
APQ	Amplitude Perturbation Quotient
AUC	Area Under the Receiver Operating Characteristic Curve
CBAM	Convolutional Block Attention Module
CNN	Convolutional Neural Network
CV	Cross-Validation
DDA	Average Absolute Difference of Differences of Amplitudes
DDP	Average Absolute Difference of Differences of Periods
DFA	Detrended Fluctuation Analysis
DL	Deep Learning
FPR	False Positive Rate
FT-Transformer	Feature-Tokenizer Transformer
GAP	Global Average Pooling
GELU	Gaussian Error Linear Unit
HNR	Harmonics-to-Noise Ratio
IQR	Interquartile Range
MLP	Multi-Layer Perceptron
NH	Noise-to-Harmonics
NIDDK	National Institute of Diabetes and Digestive and Kidney Diseases
PPE	Pitch Period Entropy
PPQ	Period Perturbation Quotient
RAP	Relative Amplitude Perturbation
ReLU	Rectified Linear Unit
RFE	Recursive Feature Elimination
ROC	Receiver Operating Characteristic
RPDE	Recurrence Period Density Entropy
SMOTE	Synthetic Minority Over-sampling Technique
TNR	True Negative Rate
TPR	True Positive Rate
UCI	University of California, Irvine
